# Sequential Effects on Reaction Time Distributions: Commonalities and Differences Across Paradigms

**DOI:** 10.5334/joc.395

**Published:** 2024-09-03

**Authors:** Anne Voormann, Jeff Miller

**Affiliations:** 1 Department of Psychology, University of Freiburg, DE; 2 University of Otago, NZ

**Keywords:** sequential effect, cognitive modelling, response time distributions

## Abstract

A common finding across numerous response time (RT) paradigms is that the mean RT in one trial depends strongly on the characteristics of the immediately preceding trial. Although such sequential effects have usually only been considered within each single paradigm in isolation from the others, there are important similarities across paradigms between the theoretical accounts of these effects. However, so far there has been no systematic comparison of sequential effects across paradigms. To investigate the possible relationships between sequential effects in different paradigms, we conducted an experiment examining sequential effects in visual search, two-choice RT, interference, and task-switching paradigms, using methods designed to maximize the similarity of stimuli and responses across paradigms. Detailed analyses of the observed RT distributions were carried out using both descriptive (e.g., ex-Gaussian) and process-oriented (e.g., diffusion models) methods. The results reveal significant empirical similarities and differences between the sequential effects observed across different paradigms, and in some cases even across different conditions within a single paradigm. Furthermore, the sequential effects are more similar to one another for some pairs of paradigms than for others. These results imply that some cognitive processes eliciting sequential effects are shared across paradigms while others seem to be paradigm-specific.

People change their behaviour from moment to moment, not just randomly, but also—and perhaps mainly—in response to their recent environments and experiences. Many effects of such hysteresis on decision-making and performance have been documented by showing sequential dependencies across a wide variety of research areas, including visual (Ceylan & Pascucci, 2023), auditory (John, 1973), and multi-sensory judgment tasks (Irwin & Preston, 1937), two-choice reaction time (2CRT) tasks (Bertelson, 1963; Kornblum, 1973; Rabbitt & Vyas, 1979), visual search tasks (Rabbitt et al., 1977), and many others. Thus, there is ample evidence that trial-to-trial variation in performance is not entirely random but is at least partly driven by such momentary changes in the underlying cognitive systems involved in task performance.

Often, stochastic models of respone time (RT) performance in choice tasks have ignored such hysteresis, assuming for simplicity that the decision-maker is in the same state (or in one randomly chosen member from the same set of potential states) at the beginning of each trial (Brown & Heathcote, 2008; McClelland, 1979; Miller & Ulrich, 2003; Ratcliff & Murdock, 1976; Usher & McClelland, 2001). These models provide useful first approximations for the investigation of decision making, but in order to be complete models they must ultimately “explain the many sources of variation which affect results” (Hale, 1967, p. 133), including sequential dependencies. To refine these models further, then, it seems helpful to investigate the mechanisms underlying sequential dependency effects across a variety of paradigms.

One type of sequential dependencies are sequential effects characterized by performance (e.g., RT, accuracy) gains in the case of a repetition between trial *n* – 1 and trial *n* and performance losses in the case of an alternation (e.g., Soetens et al., 1985). And as introduced above, this same basic phenomenon occurs not only within one specific area of cognition but can be seen as a fundamental aspect of many different sub-disciplines including 2CRT (e.g., Jones et al., 2013; Soetens et al., 1985), visual attention (e.g., Found & Müller, 1996), cognitive control (e.g., Gratton et al., 1992; Schmidt, 2013), and task-switching (e.g., Jersild, 1927; Kiesel et al., 2010) paradigms, to name just a few.

In 2CRT paradigms, for example, sequential effects arise at the level of response categories. In these tasks, participants are required to categorize each presented stimulus in terms of two previously defined categories. For example, participants could have to indicate whether an array of dots has a majority of red or green dots. In those tasks, a very common observation is that a response in trial *n* is on average faster and more accurate in case of a repetition of the stimulus and response category from trial *n* – 1 as compared with an alternation of the stimulus and response category (Bertelson, 1963; Rabbitt & Vyas, 1979; Soetens et al., 1985).

In the area of visual attention, sequential effects are mostly referred to as inter-trial effects. These occur, for example, in a simple pop-out search task (Krummenacher & Müller, 2012). In such a task, participants respond to the absence or presence of a target stimulus among homogeneous distractor stimuli. The target differs from the distractors only in one dimension, for example, its form or its color. Sequential effects in these tasks arise mostly on the level of the target’s distinguishing dimension (Müller et al., 1995). In the case of a dimension repetition between trial *n* – 1 and trial *n* (e.g., a color dimension repetition with a blue target among green distractors in trial *n* – 1 and a red target among green distractors in trial *n*), responses are on average faster compared to a dimension alternation (e.g., a blue square target among green square distractors in trial *n* – 1 and a green circular target among green square distractors in trial *n*).

In interference tasks, sequential effects are not bound to stimulus features but instead arise at the level of stimulus congruence. Stimuli in interference tasks, like the flanker task (Eriksen & Eriksen, 1974), the Simon task (Simon, 1969), the prime-probe task (Neumann & Klotz, 1994), and the Stroop task (Stroop, 1935), all include not only a response-relevant dimension but also a response-irrelevant dimension that may match or conflict with the response-relevant dimension. A common phenomenon in these tasks is the congruency effect, which describes the performance difference between stimuli in which both dimensions are associated with the same response (congruent stimuli) and those in which the two dimensions are associated with different responses (incongruent stimuli). Sequential effects in interference tasks occur at the level of these stimulus types. Repetitions of congruent or incongruent stimuli are associated on average with performance gains. In contrast, alternations from a congruent stimulus to an incongruent one or vice versa elicit performance losses (Mayr et al., 2003).^1^

Finally, in task-switching studies sequential effects arise at the level of repetitions versus alternations of the task that the participant is required to perform. In these studies, the stimuli of two different tasks (mostly 2CRT tasks) are intermixed within each block, and participants are required to keep both task sets in mind and select the correct response from a particular task set indicated by, for example, a task cue. In these paradigms a common observation is that the repetition of a task in two successive trials leads on average to a performance improvement compared to an alternation of tasks, with the difference often referred to as task-switching costs (e.g., Jersild, 1927; Kiesel et al., 2010).

As can be seen, the behavioral pattern that emerges at the level of mean RTs and accuracy is fairly similar across paradigms, with faster and more accurate responses when something repeats across trials than when it switches. Although these observed behavioral patterns seem quite similar, the different sequential effects have been investigated—so far—mostly within their respective sub-disciplines (but see Frings et al., 2020, for a first approach to combine several sequential effects in one theoretical framework). No doubt this is partly because the trial features used to categorize the sequential relationships (i.e., response, target dimension, congruency, task) are quite different across the different paradigms, making it difficult to study them all within the same series of experiments. In addition, the different types of sequential effects have been identified in very different experimental paradigms and—at least to some extent—in very different historical contexts of RT research (e.g., Bertelson, 1963; Rogers & Monsell, 1995). In any case, ultimately it is questionable whether separate investigations of the different sequential effects are still appropriate or whether sequential effects show more similarities than only on the level of mean RTs and accuracy, and in particular whether stochastic RT models can account for the different types of sequential effects with similar mechanisms. The present experiments investigated this question using stimuli, responses, and tasks chosen to be as similar as possible across paradigms.

## Theoretical accounts of sequential effects

A first step for deciding whether it is worthwhile to investigate the sequential effects of different paradigms jointly might be to compare their theoretical frameworks. Sharing certain theoretical aspects might already hint at some similarities between sequential effects apart from their similar behavioral patterns in mean RTs and accuracy. Interestingly, although the theories for the different sequential effects studied here were developed independently, they do share some commonalities.

### The role of expectations

First, the role of bottom-up versus strategic influences in producing sequential effects has been considered in all of the paradigms examined here. In visual search, for example, there is a debate about the extent to which sequential effects are elicited by strategic top-down weighting of visual dimensions (e.g., color, form) which speeds target detection when the expected dimension matches the actual presented target dimension. Whereas the dimension weighting (Found & Müller, 1996) and guided search 2.0 (Wolfe, 1994) accounts assume that such a strategic top-down modulation does speed visual search with dimension repetitions, the feature priming account (Maljkovic & Nakayama, 1994) assumes that the faster search in repetition trials is driven only by automatic feature and dimension priming without any influence of strategic processes. Furthermore, some accounts postulate a mixture of automatic and strategic processing. For example, the stimulus-driven capture account (Theeuwes, 2010, 1993) assumes that stimuli are captured only based on their automatically-determined salience, although strategic top-down processes can operate at the level of stimulus processing.

While for the visual search paradigm the discussion about bottom-up and strategic influences takes place on the level of stimulus processing, for the 2CRT paradigm it is generally thought that the response-stimulus interval (RSI) determines whether responses are influenced more by automatic facilitation or by strategic expectations. For short RSIs, automatic facilitation speeds repetition responses, whereas for long RSIs the response mode shifts towards the use of strategic expectations about the upcoming stimulus which might even elicit an alternation advantage (Soetens et al., 1984, 1985).

A similar observation has been made within task-switching paradigms. Here, task-switching costs decrease with increasing cue-to-stimulus interval (CSI). Although some authors (e.g., Mayr & Kliegl, 2003; Rogers & Monsell, 1995) argue that this decrease results from an active strategic preparation process in which task-sets are pre-activated to prepare for the upcoming task, other authors (e.g., Allport & Wylie, 2000) argue that task-switching costs occur simply because previous task-sets are still activated with short CSIs, and this activation automatically interferes with the new upcoming task-set.

Automatic interference between stimulus dimensions also plays a major role in models for responses in interference tasks. According to the prominent conflict monitoring model (Blais et al., 2007; Botvinick et al., 2001), this interference elicits a conflict that is used strategically to adapt the amount of cognitive control implemented in the subsequent trial. Higher levels of cognitive control reduce the influence of irrelevant dimensions, leading to smaller congruency effects following trials in which conflict was present. An opposing view assumes, however, that sequential effects in interference tasks occur simply because of contingency learning and the automatic activation of previously stored stimulus-response bindings from episodic memory that facilitate the retrieval of the correct response (Hommel, 1998; Schmidt & De Houwer, 2011).

### The contribution of memory

Second, the learning of contingencies and the retrieval of traces stored in episodic memory are also considered to be important sources of sequential effects in a number of paradigms. As mentioned in the previous paragraph, the storage and retrieval of stimulus-response bindings is one of the most prominent accounts of these effects in interference tasks (Dignath et al., 2019; Hommel, 1998).

A similar approach has also been taken for visual search. For example, Huang et al. (2004) assume that feature and dimension repetition gains are elicited through automatic retrieval of previously stored episodic memory traces. These traces facilitate performance in the case of a match with the features and dimensions in the current trial, and they harm performance if there is a mismatch.

The same rational has also been applied with respect to binding and retrieval in action control (Frings et al., 2020), leading to the only theoretical framework that tries to account for sequential effects from multiple paradigms. This framework assumes that the binding of the stimulus, the given response, and any subsequent effects of that response into an event file takes place on every trial. In the case of a match between features of the current situation with features from the previous event file, the earlier event file can be reactivated and thereby facilitate the response selection. If the current trial’s event file features mismatch those of the previous event file, however, performance is harmed.

Instead of the storage of whole events, within the 2CRT task there are also more parsimonious accounts that assume only the implicit learning of the base rate and the repetition rate of the stimuli which help to adapt expectations for the current trial (Cho et al., 2002; Goldfarb et al., 2012; Jones et al., 2013). Those accounts are especially helpful when considering not only the effects from the previous trial *n* – 1 on the current trial *n* but also when considering the effects on the current trial of multiple previous trials, *n* – 1, *n* – 2, and *n* – 3.

### The influence of cognitive control

A third shared commonality among the accounts of sequential effects in different paradigms considers the detection of conflict and the performance adaptations carried out in response to its presence. As mentioned earlier, the detection of conflict between response-relevant and response-irrelevant stimulus dimensions and the subsequent adjustments of cognitive control to improve performance are a very prominent explanation for the occurrence of sequential effects in interference tasks (Botvinick et al., 2001, 2004). For interference tasks, even the conflict elicited by a stimulus might be stored in an event file in episodic memory and retrieved in a subsequent trial (Dignath et al., 2019).

Furthermore, other forms of conflict have also been proposed for other paradigms. For example, the conflict between a previously-activated task-set and a new, to-be activated task-set is considered to contribute to task-switching costs (Allport & Wylie, 2000). Even an alternation between stimuli from trial *n* – 2 to *n* – 1 in 2CRT tasks seems to elicit conflict and a subsequent control adaptation. This evokes an effect called alternation-based interference, which is the slowing of responses after a previous alternation compared to a previous repetition in 2CRT tasks (Jentzsch & Leuthold, 2005).

### Interim summary of theoretical accounts

As can be seen from this short review, the theoretical accounts of sequential effects in different paradigms do share some assumptions concerning the role of expectations, the contribution of (implicit) memory, and the influence of cognitive control, although not all assumptions have been considered within all paradigms (e.g., conflict is rarely considered in visual search). Nevertheless, based on the reviewed theories it is still difficult to decide whether it might be fruitful in future research to focus on the commonalities among different sequential effects jointly, as has been done within the framework of binding and retrieval in action control (Frings et al., 2020), or whether it is more appropriate to investigate the sequential effects from different paradigms separately to acknowledge their distinctness.

## Advantages of investigating RT distributions

Apart from commonalities in their theoretical assumptions, one approach to deciding whether paradigms elicit similar effects is to investigate their RT distributions (cf. Fitousi & Azizi, 2023, for a similar approach investigating global/local effects). So far, sequential effects in most reviewed paradigms have mainly been investigated with respect to their mean RT and accuracy. However, important information about cognitive processes may get lost when considering only mean RTs instead of their full distribution (see, e.g., Balota & Yap, 2011; Heathcote et al., 1991; Matzke & Wagenmakers, 2009; Van Zandt, 2000; Whelan, 2008). Furthermore, similar changes on the level of mean RTs can be caused in various ways (Spieler et al., 2000), and possible effects that occur at the level of the RT distribution might be masked when only considering the distributional mean (Heathcote et al., 1991). Therefore, a stronger test for comparing sequential effects across paradigms is to consider changes in the distributions instead of relying only on changes in means.

Investigating the changes in sequential effects at the level of their RT distributions may help to decide whether it would be useful to investigate sequential effects in multiple paradigms together, because these changes might point to the existence of common cognitive mechanisms that are shared across paradigms. For example, to the extent that the sequential effects in different paradigms are brought about by a common cognitive mechanism, one would expect that this commonality is reflected in analogous changes in the RT distributions of repetition versus alternation trials. However, to the extent that the distributional differences between repetitions and alternations differ substantially across paradigms, then a common cognitive mechanism seems less plausible.

Thus the aim of the present study was to investigate the nature of the sequential effects on RT distributions across different paradigms. To the extent that the distributional effects are similar between paradigms, as they have already been found to be in mean RTs, this should encourage future research to look more deeply into those paradigms and explore further the existing between-paradigm commonalities in theoretical accounts of sequential effects. On the other hand, the results might also help to clarify differences between paradigms in the underlying causes of their sequential effects. In that case, the present study would emphasize the need for future studies to pursue those theoretical explorations separately from one another.

Two different approaches can be applied to assess differences between RT distributions. On the one hand, it is possible to use a rather descriptive approach to describe these differences. The distributions can be described, for example, in terms of estimates of RT distributions pooled across participants (e.g., Vincentizing, Vincent, 1912, or percentile rank pooling, Miller, 2021) or fitted ex-Gaussian distributions (e.g., Hohle, 1965). Other approaches aim to describe RT distributions through explicit models of psychological processes such as the diffusion model (Ratcliff, 1978). Such models could suggest specific, parameter-based interpretations of the processes eliciting the distributional changes associated with sequential effects.

### Vincentizing

One of the simplest methods of examining pooled RT distributions—and examining effects of experimental factors upon them—is that of “Vincentizing” (Vincent, 1912). First, using standard quantile point estimation techniques (e.g., Gilchrist, 2000), researchers estimate a set of quantile points (e.g., 5th, 15th, 25th, …, 95th) within the observed RT distribution of each participant in each condition. Second, for each condition and quantile point separately, the average of these estimates across participants is taken as the estimated value of the quantile point in the group distribution for that condition (for further details see, e.g., Jiang et al., 2004; Ulrich et al., 2007; Van Zandt, 2002). It is known that the group distributions estimated in this way do not necessarily preserve the mathematical shapes of the individual-participants’ RT distributions (e.g., Sternberg, 2023; Thomas & Ross, 1980). Shape distortions may be especially problematic with some distributions that are commonly used as models for RT (e.g., ex-Gaussian, Weibull; Rouder & Speckman, 2004). Nevertheless, Vincentizing is a simple and common method of distribution averaging, and it does provide a convenient, easily visualized depiction of the between-condition differences in cumulative RT distributions, as is illustrated in Figure 3.

### Percentile rank pooling

Percentile rank pooling (Miller, 2021) is a flexible non-parametric method which is especially helpful for the inspection of distributions from conditions with a low number of trials or for a comparison of distributions that differ in their scale (e.g., distributions with a different onset and variance of RTs). Its computation is based on a simple two-step logic. In a first step percentile ranks (PR) are computed for the RT of each trial separately within each participant but *across multiple to-be-compared experimental conditions*. The ranks are defined as


1 \[
RT_{\rm pr}(t)=\frac{L+0.5\cdot E}{N}
\]

where *t* describes the value of the RT, *L* expresses the number of trials across the to-be-compared conditions with RTs less than *t, E* defines the number of trials with RTs equal to *t*, and *N* represents the total number of trials (Miller, 2021). In a second step, the trials from each of the conditions under investigation are selected and their computed percentile ranks \[
RT_{\rm pr}(t)
\] are pooled across participants separately for each of the conditions. As a result, one obtains the density or frequency (vertical axis) of each percentile rank (horizontal axis) separately for each of the conditions being compared (color). Figure 4 depicts an example of the resulting plots.

There are three characteristics of percentile rank pooling that make the method a useful option for comparing RT distributions. First, it aims to compare the full distributions of RTs in different conditions rather than just differences in a pre-specified set of quantiles or bins. Second, because percentile rank pooling does not rely on means or medians computed within each condition separately as are often used with other popular methods such as quantile averaging (Ratcliff, 1979) or bin averaging (De Jong et al., 1994), its results are more stable for conditions in which there are only a few trials per participant. Third, because percentile rank pooling considers a non-parametric transformation of RTs to percentile ranks, it simplifies the comparison of between-participant and even between-experiment conditions that might typically operate on different scales, for example, RT distributions with different means and variances. These characteristics make percentile rank pooling attractive for the present purpose of comparing repetition effects on full RT distributions across different paradigms.

### Ex-Gaussian

Another way to describe RT distributions is by means of the parameters of theoretical distributions that match the shapes of RT distributions well. Hohle (1965) observed that RT distributions show similarities with an ex-Gaussian distribution. An ex-Gaussian distribution is an exponentially modified Gaussian distribution which is mathematically described as a convolution of independent exponential and normal components. Therefore, three parameters describe the distribution. *τ* specifies the mean of the exponential component, which reflects the length of the distribution’s upper tail and is thus responsible for its skew. The normal component is specified by *μ* and *σ*, which reflect the location and width of the distribution’s lower tail. The mean and the variance of the ex-Gaussian distribution are characterized as


2 \[
E(X)=\mu +\tau
\]

and


3 \[
Var(X)=\sigma ^{2}+\tau ^{2}.
\]

Previous studies demonstrated that the ex-Gaussian distribution describes RT distributions very well (Luce, 1986) and can even uncover experimental effects that are hidden if one would only consider differences in mean RT. For example, Spieler et al. (2000) demonstrated that—although all interference paradigms showed facilitation of mean RT in congruent relative to incongruent trials—the effects on the parameters of the ex-Gaussian distribution can differ between paradigms. Specifically, whereas congruency influenced the normal component for all paradigms, it only influenced the exponential component for some paradigms (e.g., the Stroop task and the global-local-task).

Based on the observation that some effects are only captured in certain components of the ex-Gaussian distribution, it is tempting to interpret these components in terms of psychologically processes. Hohle (1965) suggested that the normal component captures mainly motor processes while the exponential component might reflect the time needed for the decision itself. However, more recent studies have demonstrated that such a clear distinction does not hold either in simulation-based analyses (Matzke & Wagenmakers, 2009) or experimentally (Rieger & Miller, 2020). Nevertheless, the ex-Gaussian distribution is a very useful tool to get a more detailed description of RT data (Balota & Yap, 2011; Whelan, 2008), even though one must be cautious about interpreting changes in parameter values as reflecting effects on specific underlying processes (Heathcote et al., 1991; Matzke & Wagenmakers, 2009; Rieger & Miller, 2020).

### Diffusion models

#### Four-parameter diffusion model

**Figure 1:**
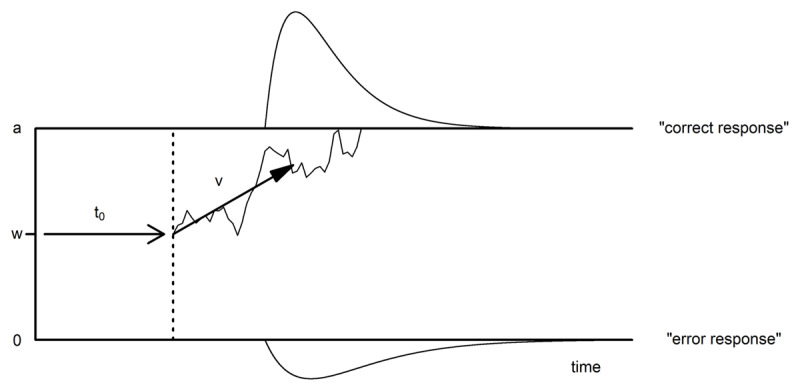
Schematic representation of an accumulation process towards the correct response in a four-parameter diffusion model with *v* as drift rate towards the boundary for a correct response, *w* the relative starting point of the accumulation process, *a* representing the boundary separation, and *t_0_* the time for stimulus encoding and response execution. The distribution on the upper boundary represents the RT distribution of correct responses and the distribution on the lower boundary depicts the RT distribution of errors.

In contrast to the two previous approaches, the diffusion model (Ratcliff & Murdock, 1976) is based on a specific psychological model of the decision-making process, so changes in its parameter values necessarily have specific psychological interpretations, as has been illustrated in numerous binary decision RT tasks (e.g., Goldfarb et al., 2012; Klauer et al., 2007; Schmitz & Voss, 2014; Schwarz, 1993). This model assumes that response decisions are based on a diffusion process accumulating evidence over time with a mean drift rate *v*, which represents task easiness or task-related ability (see Figure 1; Ratcliff et al., 2015). Once the accumulated evidence reaches one of two response criteria that are separated by *a* units, a response is elicited. However, the decision process can be biased towards one of the two response alternatives which is captured by the relative starting point *w* of the accumulation process, where *w* = .5 describes an unbiased accumulation process. In addition to these parameters describing the decision time, the final RT includes an additional non-decision time, *t_0_*, which reflects the time needed for stimulus encoding and response execution (Ratcliff & McKoon, 2008). Those four parameters represent the main parameters of the diffusion model and in the following paragraphs we will refer to a diffusion model considering only these four parameters as the ‘four-parameter diffusion model’.

#### Seven-parameter diffusion model

To account for different RT distributions for correct responses and error responses as well as for the observation of fast error responses, Ratcliff & Tuerlinckx (2002) introduced trial-to-trial variability in some parameters. For example, across trials the drift rate is considered to be normally distributed with standard deviation *σ*_v_. Furthermore, trial-to-trial variation in the response bias as well as varying times for stimulus encoding and response execution is reflected by a uniform distribution of the starting point of the accumulation process with range *σ_w_* and by a uniform non-decision time with range \[
\sigma_{t_{0}}
\]. In the following paragraphs we will refer to this more complex version of the diffusion model as the ‘seven-parameter diffusion model’.

Given that the diffusion model has passed a number of experimental tests (see, for example, Ratcliff & McKoon, 2008; Voss et al., 2004), some studies have already considered it to describe the RT differences between repetition and alternation trials. For example, Burnham (2018) applied the diffusion model to a feature search task and observed that the drift rate was increased and the non-decision time was decreased in repetition trials relative to alternations. Similarly, in a task-switching paradigm Schmitz & Voss (2012) found that the task sequence had an impact on the drift rate, with a higher drift rate after repetition trials compared to alternation trials. In both studies, however, the authors used a six-parameter version of the diffusion model assuming unbiased decisions with the relative starting point fixed at *w* = .5. That assumption is questionable, especially because Goldfarb et al. (2012) demonstrated that for 2CRT tasks a biased starting point that depended on the repetition rate described higher-order sequential effects quite well. Therefore, Goldfarb et al. (2012) introduced a variant of the four-parameter diffusion model in which the starting point depended on the actual sequence of repetitions and alternations in the previous trials with the exact number of relevant previous trials being estimated. This introduced a bias toward either a repetition response or an alternation response and was well able to mimic the observed RTs and error rates in second-order sequential effects (i.e., considering the history of the previous three trials). Thus, despite the existence of previous research examining repetition effects on diffusion model parameters within two tasks, the nature of these effects is still unclear. The discrepant results can either be the result of different model variants considered in the different studies or can hint towards differences in the effect of repetitions versus alternations on diffusion model parameters in different paradigms. Thus, to gain further evidence concerning this effect, in the present study we compared the repetition versus alternation trials of various paradigms using both the four- and seven-parameter versions of the diffusion model.

#### EZ-Diffusion model

Accurate estimation of the parameters of the seven-parameter and the four-parameter diffusion model requires quite a large number of trials, including many error responses, and it can be computationally challenging from time to time (Wagenmakers et al., 2008). To circumvent these limitations, Wagenmakers et al. (2007) developed the EZ-diffusion model (EZ) which represents a mixture between typical descriptive analyses based on means and full distributional descriptions. In contrast to the four- and seven-parameter diffusion model which consider the whole response distributions for parameter estimation, the EZ model needs only the RT means and variances of correct responses, and accuracy levels in each condition. In the original form of the EZ model, these variables are used to retrieve the psychologically most relevant parameters of the seven-parameter diffusion model via closed form relationships—namely, the drift rate *v*, the boundary separation *a*, and the non-decision time *t_0_* (Wagenmakers et al., 2007). To allow decisions to be biased towards one of two responses, Grasman et al. (2009) introduced an extension of the EZ model which also allows estimation of the relative starting point *w* of the accumulation process by considering an additional aspect of the results: the mean RT of incorrect responses.

Thus, the attraction of the EZ model lies in its simplicity. Although some argue that it is too simple and, therefore, especially vulnerable to attentional lapses, fast guesses, and other sources of abnormal processing (Ratcliff, 2008), it provides an accurate description even for data simulated from the seven-parameter diffusion model (van Ravenzwaaij & Oberauer, 2009), and its parameters have been found to be affected by experimental manipulations in psychologically plausible ways (convergent validity; Arnold et al., 2015). Because of its robustness in case of low error rates as well as its easy application, the model seems to be an excellent supplement to the seven-parameter and four-parameter diffusion model in our case because the error rates are typically low in the paradigms we selected.

### Present study

To obtain a more comprehensive view of the changes in RT distributions between repetition and alternation trials, in the present study we applied multiple analysis methods to data from four paradigms: a visual search paradigm, a 2CRT paradigm, an interference paradigm, and a task-switching paradigm. Therefore, we collected data from all four paradigms (online) utilizing similar stimuli in all paradigms to keep the task requirements as comparable as possible between paradigms. Specifically, we used a pop-out search task for the visual search paradigm, a color-majority task for the 2CRT paradigm, a color-flanker task for the interference paradigm, and both a color-majority and a form-majority task for the task-switching paradigm. In a next step we applied both descriptive distributional analyses (i.e., Vincentizing, percentile rank pooling, and ex-Gaussian distribution fitting) as well as model-based distributional analyses (i.e., seven-parameter, four-parameter, and EZ diffusion models) to these data to investigate whether the repetition versus alternation between dimensions, categories, congruency, and tasks produced similar or different changes in the various analyses’ descriptors of performance in these tasks.

## Method

All files to run the experiment, the participants’ data as well as the analysis scripts are available on the open science framework (https://osf.io/p6v9z/).

### Participants

We were interested in at least medium-sized effects (*d*_*z*_ = 0.5). When considering in G∗Power (Faul et al., 2009) a counterbalanced design between conditions and two-tailed testing with an *α* = .05 and a power of .95, this leads to a required sample size of *n* = 56 per paradigm. Participants were recruited via prolific, a platform for online studies. We restricted our participant pool to people who were between 18 and 45 years old, spoke German fluently and as a first language, had normal or corrected visual acuity with no deficits in color vision, and participated on a laptop or PC. For participation, participants received a monetary compensation of 4£. We included in the analyses only those participants with at least 70% correct responses, participants whose arcsine transformed error rates deviated at most three SDs from the sample mean of arcsine transformed error rates, and participants whose mean log RT deviated less than or equal to three SDs from the sample mean log RT. This led to final sample sizes of 56 participants within the visual search, 2CRT, and task-switching paradigms, and to 57 participants within the interference paradigm. The samples seemed comparable in the four different paradigms in terms of age and gender. In the visual search paradigm age ranged from 18 to 45 years with a mean of 29.0 years (*SD* = 6.5), with 24 male, 30 female, and two diverse participants. In the 2CRT paradigm age ranged from 20 to 45 years with a mean of 29.1 years (*SD* = 6.7), with 17 male, 37 female, and two diverse participants. In the interference paradigm age ranged from 18 to 43 years with a mean of 29.8 years (*SD* = 7.2), with 23 male, 33 female, and one diverse participant. In the task-switching paradigm age ranged from 19 to 45 years with a mean of 27.8 years (*SD* = 6.7), with 17 male, 37 female, and two diverse participants.

### Design

We conducted an online study using paradigm as a between-subject factor. Within each paradigm, there were additional paradigm-dependent within-subject factors, including repetitions versus alternations of the type studied within each paradigm.

### Materials and Procedure

The stimuli in all paradigms consisted of a 9 × 9 square grid of colored shapes that had the size of half of the screen height, which appeared on a white background. Each object in the array had a size of 2% of the screen height. The objects’ positions were defined by equally-spaced grid points. However, in order to avoid a symmetric pattern, we jittered each object’s position on the horizontal and vertical axis by a random offset between 0 and 2% of the screen height. The exact color and form of the objects varied between the different paradigms.

#### Visual search paradigm

In the visual search task, we distinguished between two types of stimulus displays: target present versus absent. In target-absent trials, the object array consisted only of distractor objects that were green squares. In target-present trials, one target object was present among the distractor objects. The target object differed from the distractors in only one feature dimension, either its color or its form. Therefore, targets could be either red or blue squares or green triangles or circles. The target position was constrained such that it never appeared in the center of the array or as an outer object.

In each block there were 40% target-absent trials and 60% target-present trials, which appeared in random order. Each target appeared equally frequently within each block. On each trial, the participants’ task was to indicate the presence or absence of a target stimulus.

#### 2CRT paradigm

In the 2CRT paradigm we also distinguished between two types of stimuli, those with a majority of red objects versus those with a majority of green objects. The presented objects were all squares but each square could be either red or green. In majority green trials, 57 of the 81 objects were colored green while the remaining 24 were red. In majority red trials these color proportions were reversed. The participants’ task was to indicate the majority color in each trial. Majority red and majority green trials appeared equally frequently within each trial block, with their order being random.

#### Interference paradigm

In the interference task participants were instructed to respond to the color of the central square within a 9 × 9 grid of square objects, and we distinguished between congruent and incongruent trials. In congruent trials all objects had the same color, whereas in incongruent trials the central object had a different color than all of the other flanking objects.

We considered a confound-minimized design, which means that we used different object colors in odd and even trials to avoid exact and partial stimulus repetitions (Kunde & Wühr, 2006). In odd trials objects were either red or green and in even trials objects were either orange or blue. In each block, each color-trial-type combination (e.g., red-congruent or blue-incongruent) appeared equally frequently.

#### Task-switching paradigm

In the task-switching paradigm we considered two different tasks, a majority-color task and majority-form task. The majority-color task was identical to the 2CRT task in both composition of the stimuli and the required response. In the majority-form task all objects were blue, but they were a mixture of triangles and circles. In majority-triangle trials 57 of the 81 objects were triangles and the remaining 24 were circles. In majority-circle trials these form proportions were reversed.

In each block, each task and each stimulus type (e.g., majority green, majority circle) appeared equally frequently. The task could be inferred from the stimulus; for example, the objects were all the same color in majority-form task trials but not in majority-color task trials. In addition, a colored frame around the object array highlighted the type of the task in each trial. The majority-color task was indicated by a black frame and the majority-form task was indicated by an orange frame. Depending on the presented task, participants had to indicate either the majority color or the majority form.

#### General procedure

After giving online informed consent and demographic information, participants were randomly assigned to one of the four paradigms. The study consisted of 10 experimental blocks with an additional practice block that included fewer trials but was otherwise identical in its procedure to the experimental blocks. Each experimental block consisted of 122 trials, and the practice block consisted of 22 trials with the first two trials of each block serving as warm-up trials. Warm-up trials were chosen randomly from the stimulus pool of the respective paradigm; however, they were selected in such a way that the critical balancing of each paradigm remained unaffected (i.e., one target present and one target absent trial for visual search, one majority red and one majority green trial for the 2CRT task, one congruent and one incongruent trial for the interference task, and one majority-color and one majority-form trial for the task-switching paradigm). Thus, there was always an alternation of the sort studied in each paradigm between trials 1 and 2.

Each block started with a countdown of the numbers 3, 2, and 1 (800 ms per number) preceding the first trial. Each trial started with the presentation of a fixation cross in the screen center for 250 ms. This was directly followed by the stimulus that remained on the screen until a response was recorded. In practice blocks, participants got an error feedback message (5 s) which reminded them of the correct key mapping. This error feedback was removed in experimental blocks. An inter-trial interval of 450 ms separated two consecutive trials.

In all paradigms, participants were instructed to respond as quickly and accurately as possible. Because all paradigms required binary responses, participants in all paradigms were instructed to respond with the ‘F’ and ‘J’ keys on their computer keyboards. For all paradigms the mapping of keys to stimuli types (e.g., present/absent) was counterbalanced across participants. After each block, participants were informed about their mean response speed and accuracy and were allowed to take self-paced breaks. The duration of the whole experiment was approximately 40 min.

### Analysis

Within each of the four tested paradigms, we first computed ANOVAs to examine effects of the within-subject factors on mean RT to obtain an initial overview of the results. We then compared the RT distributions for repetition versus alternation trials in the four paradigms, using the above-mentioned methods. Specifically, we computed the Vincentized RTs, percentile rank values \[
RT_{\rm pr}
\], and the parameters of the ex-Gaussian distribution, the EZ model, the four-parameter diffusion model, and the seven-parameter diffusion model.^2^ We estimated the parameters separately for each participant and for repetition and alternation trials, and—because the size of the sequential effects differed depending on the stimulus type (see behavioral results)—separately for each stimulus type (e.g., target dimension for the visual search paradigm, congruency for the interference paradigm, and task for the task-switching paradigm). To obtain parameter estimates, we used maximum likelihood estimation in R (R Core Team, 2016) for the ex-Gaussian, the four-parameter diffusion model, and the seven-parameter diffusion model, and we minimized the sum of the squared differences between the predicted and observed values for the EZ model.^3^ More precisely, we used the optimizing function optim with the Nelder-Mead optimization algorithm. To minimize the possibility that the optimization function converged by chance on parameter estimates for a local maximum, we repeated the procedure 50 times for the ex-Gaussian distribution and the EZ model and 10 times for the four-parameter diffusion model and the seven-parameter diffusion model, selecting as the final estimates for each participant and condition the parameter estimates with the maximum likelihood.^4^ In addition, we compared whether the conclusions drawn would change depending on whether the best parameters were selected from *k* or only from *k* – 1 runs to ensure valid conclusions.

## Results

### Data preparation

For all analyses, we excluded trials from the practice block, the first two trials of each experimental block, post-error trials, and trials in which the RT suggested that the participant did not perform the task adequately (e.g., due to guessing or in-attention). The latter were defined by extremely fast responses (less than 300 ms in the visual search paradigm and the interference paradigm or less than 250 ms in the 2CRT paradigm and the task-switching paradigm) or extremely slow responses (RTs greater than 3000 ms). To determine these lower RT boundaries, we checked the mean accuracy and cumulative accuracy across participants for all responses within each successive 50 ms RT interval. We took the RT boundary of the first of two successive intervals that showed performance above chance level in both the absolute and cumulative accuracy as the lower boundary for trial exclusion.^5^ This led to the exclusion of 3.8% of all trials for the visual search paradigm, 4.1% for the 2CRT paradigm, 5.1% for the interference paradigm, and 5.5% for the task-switching paradigm.

The definition of alternation and repetition trials depended on the paradigm, as indicated in the introduction. For the visual search paradigm, these were defined only in the case when targets were present in both trials *n* – 1 and *n*. A trial *n* in which the target dimension changed between trial *n* – 1 and trial *n* (e.g., from the color dimension in trial *n* – 1 to the form dimension in trial *n*) was coded as an alternation, whereas a trial *n* with the same target dimension as on trial *n* – 1 was coded as a repetition. In the 2CRT paradigm repetitions and alternations refer to the stimulus and response (S-R), thus repetition trials were defined by an S-R repetition (e.g., majority green, left response) between trial *n* – 1 and trial *n*, whereas alternation trials were defined by an S-R alternation. In the interference paradigm the critical trial attribute was congruency (congruent vs. incongruent). Thus, a repetition of the congruency status of trial *n* – 1 to trial *n* was coded as a repetition trial whereas an alternation of this status was coded as alternation trial. Finally, for the task-switching paradigm a repetition of the to-be-performed task (majority-color or majority-form) between trial *n* – 1 and trial *n* was defined as a repetition trial, whereas a task alternation was defined as an alternation trial.

### Behavioral results

To check for the presence of sequential effects, we conducted a 2 × 2 within-subjects analysis of variance (ANOVA) with the factors of stimulus type in trial *n* and trial sequence (repetition vs. alternation), separately for each of the paradigms. As previously explained in connection with the distinction between repetition and alternation trials, the “stimulus type” depended on the paradigm. For the visual search paradigm, stimulus type refers to the target dimension (color vs. form target), for the 2CRT paradigm it refers to the majority color (majority green vs. majority red), for the interference paradigm it refers to the control state (congruent vs. incongruent), and finally for the task-switching paradigm it refers to the task that had to be performed (majority-color task vs. majority-form task). As the dependent variable, we included the mean RTs of trials with correct responses for each participant in each condition. Mean RTs and the corresponding values of proportion correct (PC) for all conditions are summarized in Table 1.

**Table 1:** Summary across paradigms and stimulus types of sequential effects on response time (RT) and proportion of correct responses (PC).


	*M_A_*	*M_R_*	Δ	95% CI	*M_A_*	*M_R_*	Δ	95% CI

	VS COLOR				VS FORM			

**RT**	524	479	44.7	[36.3;53.1]	645	642	2.4	[–6.0;10.8]

**PC**	.978	.993	.015	[.008;.021]	.930	.949	.019	[.008;.030]

	**2CRT**							

**RT**	584	556	36.6	[28.0;45.2]				

**PC**	.955	.969	.013	[.006;.020]				

	**INT CONGRUENT**				**INT INCONGRUENT**			

**RT**	575	562	13.5	[9.0;17.9]	608	602	5.6	[1.1;10.1]

**PC**	.961	.961	–.0002	[–.005;.004]	.940	.950	.010	[.003;.017]

	**TS COLOR**				**TS FORM**			

**RT**	712	608	103.6	[85.3;121.8]	788	697	91.6	[74.0;109.1]

**PC**	.931	.980	.048	[.038;.059]	.937	.958	.021	[.012;.031]


Note. VS = visual search paradigm; 2CRT = 2CRT paradigm; Int = interference paradigm; TS = task-switching paradigm; CI = confidence interval.

#### Visual search paradigm

**Figure 2:**
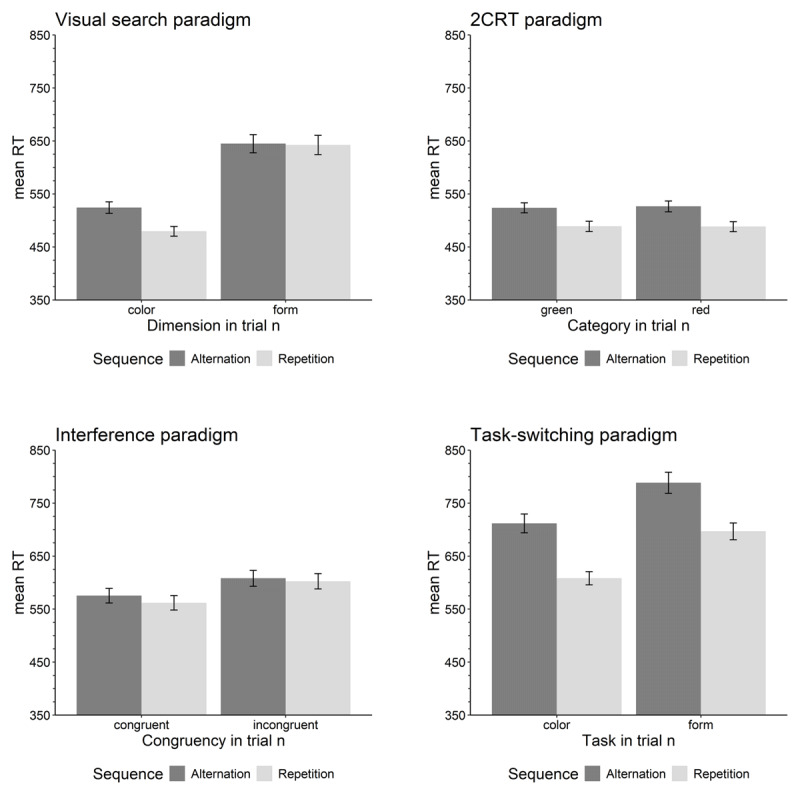
Mean response time (mean RT) in ms for repetition and alternation trials separately for each stimulus type of the four paradigms.

For the visual search paradigm, we found a significant effect of trial sequence, *F*(1,55)= 66.45, *p* < .001, \[
\eta _{p}^{2}=.547
\], with reactions being faster in repetition trials (*M* = 556 ms) compared to alternation trials (*M* = 584 ms; see Figure 2), indicating the presence of sequential effects in this paradigm. Furthermore, there was a significant effect of the target dimension in trial *n, F*(1,55)= 162.41, *p* < .001, \[
\eta _{p}^{2}=.747
\], with reactions being faster for color targets (*M* = 501 ms) than form targets (*M* = 643 ms). In addition, there was a significant interaction between the target dimension in trial *n* and trial sequence, *F*(1,55)= 48.31, *p* < .001, \[
\eta _{p}^{2}=.468
\]. A post-hoc *t*-test revealed that the sequential effect was larger for color targets (*M* = 44.7 ms) compared to form targets (*M* = 2.4 ms; *t*(55)= 6.95, *p* < .001, *d*_z_ = 1.35), and the latter sequential effect was non-significant as the 95% confidence interval included zero (see Table 1).

#### 2CRT paradigm

For the 2CRT paradigm, there was only a significant effect of trial sequence, *F*(1,55) = 71.91, *p* < .001, \[
\eta _{p}^{2}=.567
\], indicating the presence of sequential effects. Responses on repetition trials (*M* = 489 ms) were on average faster than responses on alternation trials (*M* = 525 ms). Neither the main effect of stimulus in trial *n, F*(1,55) = 0.08, *p* = .781, \[
\eta _{p}^{2}=.001
\], nor the interaction, *F*(1,55) = 0.52, *p* = .474, \[
\eta _{p}^{2}=.009
\], reached significance. As can be seen in Figure 2, responses to majority green and majority red trials were approximately equally fast and equally sensitive to sequential effects.

#### Interference paradigm

For the interference paradigm, we found a significant effect of trial sequence, *F*(1,56) = 38.60, *p* < .001, \[
\eta_{p}^{2}=.408
\], indicating the presence of sequential effects. Responses to repetition trials (*M* = 582 ms) were on average faster than responses on alternation trials (*M* = 592 ms). Furthermore, a significant congruency effect occurred, *F*(1,56) = 123.27, *p* < .001, \[
\eta _{p}^{2}=.688
\], with reactions being faster in congruent trials (*M* = 569 ms) than in incongruent trials (*M* = 605 ms; see Figure 2). Finally, there was a significant interaction, *F*(1,56) = 5.80, *p* = .019, \[
\eta _{p}^{2}=.094
\], with the sequential effect being larger for congruent trials (*M* = 13.5 ms) than for incongruent trials (*M* = 5.6 ms; *t*(55) = 2.41, *p* = .019, *d*_z_ = 0.46).

#### Task-switching paradigm

For the task-switching paradigm, we again found a significant effect of trial sequence, *F*(1,55) = 162.65, *p* < .001, \[
\eta _{p}^{2}=.747
\], indicating the presence of sequential effects in the task-switching paradigm. Responses to repetition trials (*M* = 652 ms) were on average faster than responses on alternation trials (*M* = 750 ms). Furthermore, there was a significant effect of the task in trial *n, F*(1,55) = 86.68, *p* < .001, \[
\eta _{p}^{2}=.612
\], with reactions being faster in the majority-color task (*M* = 660 ms) than in the majority-form task (*M* = 743 ms; see Figure 2). However, there was no interaction between the trial sequence and the task in trial *n* on mean RTs, *F*(1,55) = 1.70, *p* = .198, \[
\eta _{p}^{2}=.030
\], so there is no evidence that the size of the sequential effects differed between the two tasks.

### Discussion of behavioral results

Table 1 summarizes the sequential effects on RT and PC across all four paradigms, distinguishing between stimulus types for all paradigm except 2CRT, where the stimulus types did not differ. Taken together, the results on the level of mean RTs reveal that sequential effects occur within each paradigm. However, even on the level of the mean RTs it is visible in the results of the ANOVA and in Figure 2 that there exist differences in the size of sequential effects within and across paradigms. The smallest overall sequential effects occurred for the interference task, with congruent trials showing an effect of 13.5 ms and for incongruent trials of 5.6 ms. For the visual search task, the size of the sequential effect depended strongly on the target dimension. Whereas color targets showed an effect of 44.7 ms, form targets only showed a non-significant 2.4 ms effect. In contrast, the sequential effect within the 2CRT paradigm was medium-sized at 36.6 ms. Finally, the task-switching paradigm yielded the largest sequential effects, with mean effects of 103.6 ms for the color task and 91.6 ms for the form task.

These differences in the size of the mean sequential effect across paradigms might suggest that different processes contribute to their occurrence. However, finding different effect sizes within paradigms weakens the evidence for such a conclusion. As was noted in the introduction and has been demonstrated in many previous studies (e.g., Balota & Yap, 2011; Heathcote et al., 1991; Matzke & Wagenmakers, 2009; Van Zandt, 2000; Whelan, 2008), more detailed analyses of the full distributions of RTs—rather than just the means—can provide further important clues about processing differences between conditions. Therefore, we present in the next sections a systematic description of the changes in RT distributions derived from both descriptive and model-based analyses.

### Descriptive distributional analysis

#### Vincentizing

**Figure 3:**
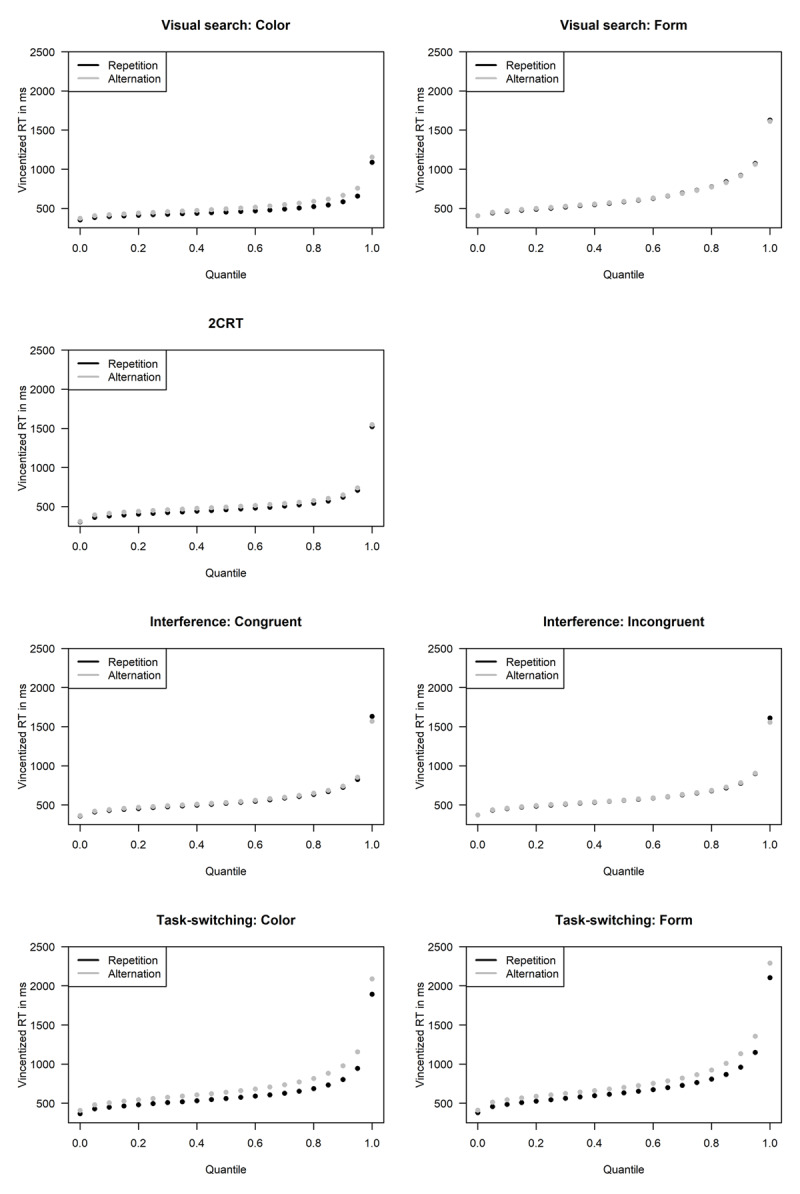
Vincentized response time (RT) per quantile for repetition and alternation trials separately per paradigm and stimulus type.

For an initial descriptive investigation of RT distributions, we used the method of Vincentizing. Therefore, we computed the RTs at the 5th, 10th, 15th, …, 95th quantiles separately for each participant’s responses in repetition and alternation trials for each stimulus type. In a next step, we averaged across participants to compute the resulting Vincentized RTs per quantile separately for repetition and alternation trials. Figure 3 depicts the Vincentized RTs per quantile separately for each paradigm and stimulus type. As can be seen for all paradigms, Vincentized RTs increase faster with quantile for the higher quantiles than for the lower quantiles, which is a consequence of the typical right-skew of RT distributions.

Additionally, for most of the graphs it can be seen that the Vincentized RTs for repetition trials are somewhat lower than for alternation trials representing the presence of sequential effects at each quantile. However, as already seen in the behavioural results, the sizes of the sequential effects differ between paradigms, as is expressed in Figure 3 by the difference between the points for repetition and alternation trials per quantile, being largest for the task-switching paradigm and smallest for the interference paradigm.

Furthermore, sequential effects seem to be strongest within different regions of the RT distributions for different paradigms. For the interference paradigm, for example, the order between repetition and alternation trials changes in the highest quantile with repetition trials showing slower RTs than alternation trials. The same pattern also occurs for the form targets of the visual search paradigm. In contrast, for the task-switching paradigm and for the color targets in the visual search paradigm, the sequential effects tend to increase with increasing quantile. And finally, for the 2CRT paradigm sequential effects seem to be relatively constant across quantiles, with only perhaps a small increase of sequential effects for the quantiles in the middle of the distribution. Thus, the Vincentized RT distributions give us a first hint that the effects of repetitions versus alternations on RT distributions do show some similarities and some differences among paradigms. However, based on these plots it is hard to draw definitive conclusions about the development of sequential effects over time, especially for the paradigms with smaller sequential effects.

#### Percentile rank pooling

**Figure 4:**
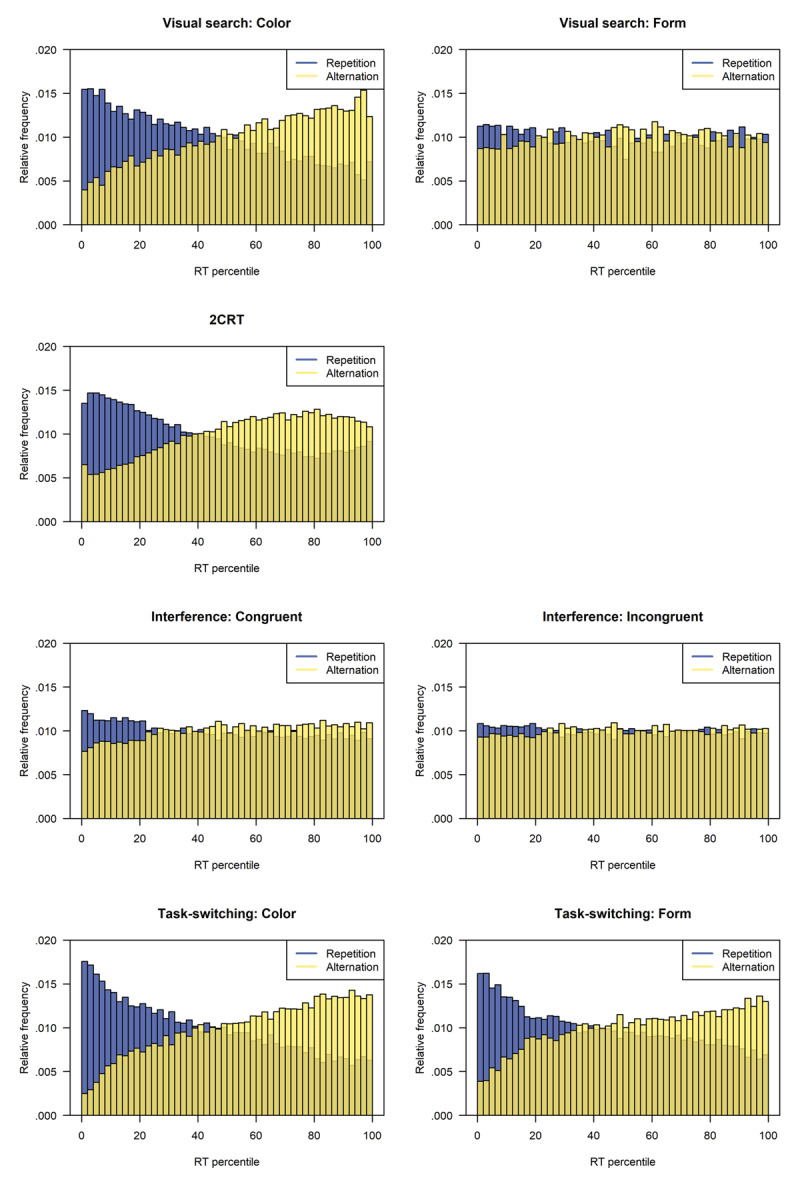
Relative frequency histograms of the pooled observed reaction time (RT) percentiles for repetition and alternation trials separately per paradigm and stimulus type.

As a second approach to investigate sequential effects on the shapes of RT distributions for each paradigm and stimulus type, we used percentile rank pooling (Miller, 2021). Therefore, in a first step, we computed the percentile rank of each RT, \[
RT_{\rm pr}
\], relative to the full set of RTs from the same participant and stimulus type (e.g., color versus form in the visual search task, congruence in the interference task). In a second step, we pooled the computed \[
RT_{\rm pr}
\] values across participants and plotted histograms of these separately for repetition and alternation trials. As can be seen in Figure 4, in all paradigms there are fewer alternation than repetition trials with low percentile ranks, and the reverse is true for high percentile ranks. This provides a more detailed view of the overall sequential effects, namely, the on-average shorter RTs in repetition trials compared to alternation trials. Furthermore, from the differences in proportions of repetition and alternation trials at the low and high ends of the histograms, one can picture the strength of the sequential effect. Strong sequential effects (i.e., large differences in proportions) are present in the visual search paradigm for the color targets, in the 2CRT paradigm, and in the task-switching paradigm, but these effects are much weaker for the form target in visual search and for the interference paradigm.

On a first view, the percentile rank pooling analysis gives a slightly different impression of the location of the sequential effect within the RT distributions than that suggested by Vincentizing. Whereas the Vincentized distributions in Figure 3 seem to suggest that the higher RT percentiles are most affected by the sequence, the pooled percentiles in Figure 4 suggest that the sequence effects are strongest on the fastest RTs. However, this impression is elicited because the two procedures use different sub-samples to depict the distributions of sequential effects. While for Vincentizing, quantiles are computed separately for repetition and alternation trials, the percentiles of the \[
RT_{\rm pr}
\] are computed over the whole RT distribution. Furthermore, it is important to keep in mind that the sequential effects for different quantiles in the Vincentized distributions are only reported descriptively. However, because the variance of the RTs varies across quantiles, one needs to be cautious in interpreting those descriptive sequential effects in terms of standardized effects.

Most important for our aim of comparing the sequential effects on RT distributions across paradigms is the shape of the \[
RT_{\rm pr}
\] histograms, and these also seem to differ. Note that in most cases there is a monotonic increase in the frequencies of \[
RT_{\rm pr}
\] values of alternation trials with increasing percentile ranks (e.g., in the visual search paradigm for color targets and in the task-switching paradigm for both tasks; see Figure 4). In contrast, in the 2CRT paradigm, it seems that the frequency of alternation-trial \[
RT_{\rm pr}
\] values initially increases with increasing percentile rank but then decreases for the highest percentile ranks. Another way to describe these differing patterns is to note that the repetition effect influences the whole RT distribution in most cases but that in the 2CRT paradigm repetition has a relatively small effect on the very slowest responses. Thus, the percentile rank pooling plots also hint towards differences between the effects of repetitions versus alternations on RT distributions among paradigms. It is still difficult, of course, to draw definitive conclusions about the mechanisms responsible for these differences based only on the percentile rank pooling plots, and it remains to be seen whether the later model-based analyses will shed any light on these mechanisms.

#### Ex-Gaussian distribution

Table 2 depicts the results of a distribution-based description of the changes in RT distributions of repetition and alternation trials, showing the mean parameter estimates for the ex-Gaussian distribution for the four different paradigms. As can be seen from the results, for all paradigms and stimulus types the *μ* parameter differed significantly between repetition and alternation trials (all *p*s < .001). Therefore, there seems to be a shift in the RT distribution with repetition trials being described on average by a lower *μ* compared to alternation trials. Furthermore, we found paradigm-specific and stimulus type-specific changes in the parameters. For the interference paradigm, for example, there was only a significant change in *μ*, but for the task-switching paradigm there seemed to be an additional change in the skew of the distribution between repetition trials and alternation trials, captured by an increase in *τ* for alternation trials compared to repetition trials (color task: *t*(55) = 7.46, *p* < .001; form task: *t*(55) = 6.95, *p* < .001). This change in *τ* also occurred for the visual search task, but only for the color targets (*t*(55) = 5.95, *p* < .001) that showed a large repetition effect. In contrast, for the form targets of the visual search task, estimates of *τ* were actually numerically smaller for alternation trials than for repetition trials, although not significantly so; this suggests that skewness was reduced in alternation trials, which would allow there to be a shift in the distribution (i.e., effect on *μ*) without a significant effect on mean RT. In the 2CRT task, on the other hand, the distributions for repetition and alternation trials also differed in the distributional width, represented by a significant change in *σ* (*t*(55) = 5.45, *p* < .001), with a larger *σ* for alternation trials than repetition trials.

**Table 2:** Ex-Gaussian distribution: Mean (M) parameter estimates for alternation (A) and repetition (R) trials, their difference (Δ), and the respective effect size with 95%-confidence interval of the standardized effects for all parameters within each paradigm and stimulus type.


	*M_A_*	*M_R_*	Δ	*d_Z_*	[LOW;UP]	*M_A_*	*M_R_*	Δ	*d_Z_*	[LOW;UP]

	VS COLOR					VS FORM				

***µ***	**415**	**393**	**21.4**	**0.42**	**[0.32;0.51]**	**454**	**442**	**12.0**	**0.21**	**[0.12;0.31]**

*σ*	22	20	2.1	0.15	[–0.22;0.52]	29	22	6.3	0.32	[–0.07;0.7]

***τ***	**113**	**87**	**26.0**	**0.46**	**[0.3;0.62]**	200	209	–9.1	0.08	[0;0.17]

	**2CRT**									

***µ***	**423**	**386**	**36.5**	**0.73**	**[0.59;0.86]**					

***σ***	**42**	**33**	**8.6**	**0.72**	**[0.43;1.02]**					

*τ*	100	103	–2.7	0.06	[–0.05;0.18]					

	**INT CONGRUENT**					**INT INCONGRUENT**				

***µ***	**442**	**430**	**12.0**	**0.18**	**[0.13;0.23]**	**465**	**461**	**4.1**	**0.06**	**[0.02;0.11]**

*σ*	37	34	2.6	0.17	[–0.07;0.41]	44	43	1.1	0.06	[–0.15;0.27]

*τ*	133	131	1.8	0.02	[–0.06;0.11]	140	139	0.8	0.01	[–0.06;0.08]

	**TS COLOR**					**TS FORM**				

***µ***	**497**	**448**	**49.2**	**0.76**	**[0.62;0.91]**	**531**	**483**	**47.5**	**0.66**	**[0.53;0.79]**

*σ*	42	37	5.1	0.19	[–0.18;0.56]	55	54	1.2	0.07	[–0.25;0.39]

***τ***	**209**	**160**	**49.4**	**0.53**	**[0.38;0.69]**	**255**	**211**	**44.0**	**0.45**	**[0.31;0.58]**


Note. Significant differences are highlighted using bold font. VS = visual search paradigm; 2CRT = 2CRT paradigm; Int = interference paradigm; TS = task-switching paradigm.

### Discussion of descriptive distributional analysis

Taken together, the patterns of sequential effects on parameters of the ex-Gaussian distributions, the \[
RT_{\rm pr}
\] histograms, and the Vincentized RTs revealed both similarities and differences in the nature of these effects on RT distributions. For the ex-Gaussian distribution, in all paradigms, *μ* was larger for alternation trials than repetition trials, reflecting a slowing of alternation responses across the whole distribution, as opposed to just a slowing of the slowest responses. This pattern can also be seen in the \[
RT_{\rm pr}
\] histograms, which show a similar tendency of fewer alternation than repetition responses in the lower percentiles for all paradigms, and in Vincentized RTs which show that repetition trials show in most conditions consistently lower Vincentized RTs than alternation responses.

However, there was increased slowing at the high end of the alternation distribution, as reflected in increased *τ* values for the ex-Gaussian distribution in the visual search and task-switching paradigms but not in the 2CRT and interference paradigms. This increase can also be seen in the Vincentized distributions, for which sequential effects increased with quantile for the task-switching paradigm and the color targets in the visual search paradigm. However, the size of the sequential effects seemed rather unaffected over quantiles for the 2CRT and interference paradigms. The change in the shapes of the RT distributions in repetition and alternation trials is also easy to see in the \[
RT_{\rm pr}
\] histograms. The frequencies of alternation RTs increased monotonically across percentile ranks for the visual search, the interference, and task-switching paradigms but not for the 2CRT paradigm. For the 2CRT paradigm, the frequencies for repetition and alternation trials seem to nearly converge at the high end of the distribution.

These distributional differences hint at differences between these two pairs of paradigms in the mechanisms underlying their sequential effects, and in particular at some process that produces especially large slowing in some alternation trials in the visual search and task-switching paradigms. Again, it will be interesting to see whether the later model-based analyses can shed any light on the nature of this process.

### Model-based distributional analyses

#### EZ model

Table 3 summarizes the parameter estimates of the EZ model and shows the parameters with significant differences between repetition and alternation trials, separately for each paradigm and stimulus type. In contrast to the ex-Gaussian distribution, there is not one specific parameter that was sensitive to the distributional differences between repetition and alternation trials for all paradigms and stimulus types, as there was no significant difference for any parameter in the case of the congruent trials in the interference paradigm. However, there was an effect on drift rate *v* for the interference paradigm for incongruent stimuli (*t*(56) = 2.14, *p* = .037). *v* was higher in repetition trials compared to alternation trials, reflecting faster evidence accumulation in the case of a repetition. An effect on *v* was also present within the other paradigms for all stimulus types (*p*s < .033).

A sequential effect on the non-decision time *t_0_* was also found in many cases, suggesting that stimulus sequence impacts not only the decision process but also the processes associated with stimulus encoding, response execution, or both. For example, a significant effect on *t_0_* occurred for the color targets in the visual search paradigm (*t*(55) = 3.57, *p* = .001), in the 2CRT paradigm (*t*(55) = 8.07, *p* < .001), and in both tasks of the task-switching paradigm (color: *t*(55) = 7.66, *p* < .001; form: *t*(55) = 5.82, *p* < .001). In all cases, this effect reflected a lower time for stimulus encoding and response execution, *t_0_*, in repetition trials compared to alternation trials.

For the color targets in the visual search paradigm, the 2CRT paradigm, and the color task in the task-switching paradigm, sequential effects were additionally evident in significant differences between boundary separation *a* (*p*s < .039) and starting point *w* (*p*s < .016) for repetitions versus alternations. For all three stimulus types, there was a higher boundary separation *a* in repetition trials compared to alternation trials. A higher boundary separation *a* is often attributed to greater response caution and less focus on speed. Larger *w* values are generally interpreted as reflecting a starting-point advantage for the accumulation of evidence towards the correct response, which also speeds this response. But the starting point *w* was closer to the correct response for repetitions compared to alternations only for the color targets in the visual search paradigm and the color task in the task-switching paradigm. For the 2CRT paradigm the pattern was reversed, so the shift in *w* actually worked against the overall RT advantage for repetitions in this case.

**Table 3:** EZ model: Mean (M) parameter estimates for alternation (A) and repetition (R) trials for all parameters within each paradigm and stimulus type as well as the respective effect size with 95%-confidence interval of the standardized effects.


	*M_A_*	*M_R_*	*d_Z_*	[LOW;UP]	*M_A_*	*M_R_*	*d_Z_*	[LOW;UP]

	VS COLOR				VS FORM			

***a***	**1.842**	**2.214**	**0.35**	**[0.01;0.68]**	1.528	1.655	0.16	[–0.11;0.43]

***v***	**3.913**	**4.881**	**0.64**	**[0.34;0.94]**	**2.265**	**2.407**	**0.2**	**[0.02;0.39]**

***t_0_***	**0.288**	**0.243**	**0.46**	**[0.19;0.72]**	0.338	0.315	0.22	[–0.01;0.45]

***w***	**0.496**	**0.499**	**0.45**	**[0.08;0.82]**	0.49	0.491	0.06	[–0.24;0.36]

	**2CRT**							

***a***	**1.135**	**1.192**	**0.2**	**[0.07;0.34]**				

***v***	**3.03**	**3.219**	**0.28**	**[0.07;0.48]**				

***t_0_***	**0.339**	**0.3**	**0.64**	**[0.47;0.81]**				

***w***	**0.505**	**0.499**	**1.08**	**[0.59;1.56]**				

	**INT CONGRUENT**				**INT INCONGRUENT**			

*a*	1.393	1.332	0.09	[–0.04;0.21]	1.248	1.273	0.05	[–0.07;0.17]

***v***	2.923	2.875	0.06	[–0.11;0.23]	**2.568**	**2.674**	**0.16**	**[0.01;0.31]**

*t*_0_	0.33	0.322	0.08	[–0.03;0.18]	0.368	0.361	0.07	[–0.06;0.19]

*w*	0.502	0.502	0.08	[–0.23;0.39]	0.506	0.505	0.21	[–0.01;0.44]

	**TS COLOR**				**TS FORM**			

***a***	**1.427**	**1.688**	**0.52**	**[0.24;0.81]**	1.591	1.588	0.01	[–0.16;0.18]

***v***	**2.021**	**2.798**	**1.2**	**[0.95;1.45]**	**1.872**	**2.199**	**0.67**	**[0.47;0.88]**

***t_0_***	**0.379**	**0.297**	**0.94**	**[0.65;1.24]**	**0.39**	**0.344**	**0.59**	**[0.37;0.81]**

***w***	**0.506**	**0.5**	**0.93**	**[0.56;1.30]**	0.503	0.503	0.03	[–0.29;0.35]


Note. Significant differences are highlighted using bold font. VS = visual search paradigm; 2CRT = 2CRT paradigm; Int = interference paradigm; TS = task-switching paradigm.

Overall, the results from the EZ model seem to reveal just as the results from the other descriptive distributional analyses that there are similarities in the distributional changes but also differences for the different paradigms and stimulus types.

#### Four-parameter diffusion model

The results for the four-parameter diffusion model are quite similar to the results of the EZ model both in their general pattern (i.e., which parameters differ for certain paradigms and stimulus types) as well as in their direction of the effects. There were only three exceptions which we will highlight here (see Table 4). First, there was no significant difference between repetition and alternation trials in the drift rate *v* for the form targets of the visual search paradigm. Instead, the starting point *w* captured those sequential effects, with the starting point being closer to the correct response in repetition trials compared to alternation trials (*t*(55) = 2.83, *p* = .006). Second, there was no effect on the non-decision time *t_0_* for the 2CRT paradigm. However, third, for the four-parameter diffusion model the starting point *w* was estimated to be closer to the correct response for repetition trials compared to alternation trials in the 2CRT paradigm (*t*(55) = 7.98, *p* < .001), which is an effect on *w* in the opposite direction compared to the results from the EZ model. All other significant effects summarized in Table 4 were in the same direction as for the EZ model, with the largest *p* amounting to .044 for the drift rate of incongruent trials in the interference paradigm.

**Table 4:** Four-parameter diffusion model: Mean (M) parameter estimates for alternation (A) and repetition (R) trials for all parameters within each paradigm and stimulus type as well as the respective effect size with 95%-confidence interval of the standardized effects.


	*M_A_*	*M_R_*	*d_Z_*	[LOW;UP]	*M_A_*	*M_R_*	*d_Z_*	[LOW;UP]

	VS COLOR				VS FORM			

***a***	**1.8**	**2.21**	**0.46**	**[0.16;0.76]**	1.48	1.6	0.21	[–0.06;0.48]

***v***	**3.7**	**4.65**	**0.73**	**[0.45;1]**	2.04	2.1	0.08	[–0.09;0.25]

***t_0_***	**0.34**	**0.32**	**0.47**	**[0.25;0.68]**	0.38	0.37	0.06	[–0.09;0.21]

***w***	**0.58**	**0.63**	**0.32**	**[0.04;0.59]**	**0.56**	**0.6**	**0.33**	**[0.09;0.57]**

	**2CRT**							

***a***	**1.47**	**1.39**	**0.36**	**[0.15;0.57]**				

***v***	**4.12**	**3.89**	**0.25**	**[0.07;0.43]**				

*t_0_*	0.29	0.28	0.17	[–0.01;0.35]				

***w***	**0.32**	**0.4**	**1.11**	**[0.76;1.46]**				

	**INT CONGRUENT**				**INT INCONGRUENT**			

*a*	1.5	1.43	0.15	[–0.06;0.36]	1.43	1.45	0.06	[–0.08;0.2]

***v***	3.39	3.39	0	[–0.11;0.11]	**3.13**	**3.27**	**0.16**	**[0.01;0.31]**

*t_0_*	0.33	0.33	0.11	[0;0.22]	0.34	0.33	0.06	[–0.07;0.19]

*w*	0.43	0.42	0.05	[–0.1;0.2]	0.39	0.39	0.01	[–0.17;0.19]

	**TS COLOR**				**TS FORM**			

***a***	**1.51**	**1.75**	**0.49**	**[0.2;0.78]**	1.71	1.7	0.01	[–0.22;0.24]

***v***	**2.4**	**3.17**	**1.03**	**[0.82;1.23]**	**2.23**	**2.6**	**0.61**	**[0.4;0.82]**

***t_0_***	**0.37**	**0.32**	**0.93**	**[0.72;1.14]**	**0.36**	**0.33**	**0.6**	**[0.32;0.87]**

***w***	**0.43**	**0.46**	**0.4**	**[0.08;0.72]**	0.42	0.42	0.02	[–0.26;0.3]


Note. Significant differences are highlighted using bold font. VS = visual search paradigm; 2CRT = 2CRT paradigm; Int = interference paradigm; TS = task-switching paradigm.

#### Seven-parameter diffusion model

The results of the seven-parameter diffusion model look a bit more diverse compared to the results of the other two diffusion models. As can be seen in Table 5, there was no significant difference between any parameter estimates of repetition and alternation trials for the visual search paradigm with form targets or with the congruent trials in the interference paradigm. For the other stimulus types and paradigms, there were significant differences between repetition and alternation trials in the drift rates *v* (*p*s < .017). In most cases the difference was characterized by a higher drift rate *v* in repetition trials compared to alternation trials; in the 2CRT paradigm, however, the pattern was reversed, with the effect on this parameter going in the opposite direction from what would be expected based on the effect on mean RT. For the color targets in the visual search paradigm and the incongruent trials in the interference paradigm, this difference in the drift rate *v* was accompanied by a significant difference in the inter-trial variability of the drift rate *σ*_v_, which was also higher in repetition trials than in alternation trials (visual search: *t*(55) = 3.54, *p* = .001; interference: *t*(56) = 2.09, *p* = .042).

Sequential effects also produced differences in the non-decision time *t_0_*. The time for stimulus encoding and response execution was higher in alternation trials compared to repetition trials for the color targets in the visual search paradigm (*t*(55) = 5.11, *p* < .001) and for both tasks of the task-switching paradigm (color task: *t*(55) = 6.23, *p* < .001; form task *t*(55) = 4.78, *p* < .001). The inter-trial variability of the non-decision time \[
\sigma_{t_{0}}
\] was also greater in alternation trials than repetition trials for the color targets of the visual search paradigm (*t*(55) = 2.61, *p* = .012) but not for either task in the task-switching paradigm.

Furthermore, the boundary separation *a* and the starting point *w* differed significantly between repetition and alternation trials for the color task in the task-switching paradigm and the 2CRT task (*p*s < .014). This time, the boundary separation *a* was higher in alternation trials compared to repetition trials, which could be interpreted as greater response caution in alternation trials compared to repetition trials. On the other hand, the starting point *w* was closer to the correct response in repetition trials compared to alternation trials and even showed a significant difference in the inter-trial variability *σ*_*w*_ for the color task of the task-switching paradigm (*t*(55) = 2.81, *p* = .007).

**Table 5:** Seven-parameter diffusion model: Mean (M) parameter estimates for alternation (A) and repetition (R) trials for all parameters within each paradigm and stimulus type as well as the respective effect size with 95%-confidence interval of the standardized effects.


	*M_A_*	*M_R_*	*d_Z_*	[LOW;UP]	*M_A_*	*M_R_*	*d_Z_*	[LOW;UP]

	VS COLOR				VS FORM			

*a*	1.45	1.61	0.23	[–0.08;0.54]	1.38	1.49	0.22	[–0.01;0.44]

***v***	**3.62**	**5.22**	**1.08**	**[0.65;1.51]**	2.31	2.34	0.02	[–0.24;0.28]

***t_0_***	**0.35**	**0.33**	**0.45**	**[0.27;0.64]**	0.38	0.38	0.05	[–0.13;0.23]

*w*	0.67	0.65	0.11	[–0.18;0.39]	0.64	0.67	0.19	[–0.04;0.43]

***σ_v_***	**0.56**	**1.19**	**0.63**	**[0.24;1.01]**	0.66	0.63	0.03	[–0.28;0.33]

\[ \sigma_{t_{0}} \]	**0.08**	**0.07**	**0.36**	**[0.08;0.64]**	0.12	0.12	0.02	[–0.22;0.25]

*σ_w_*	0.19	0.19	0.05	[–0.33;0.42]	0.24	0.25	0.12	[–0.25;0.5]

	**2CRT**							

***a***	**1.44**	**1.31**	**0.33**	**[0.14;0.53]**				

***v***	**5.93**	**5.36**	**0.49**	**[0.11;0.87]**				

*t_0_*	0.28	0.27	0.11	[–0.05;0.27]				

***w***	**0.29**	**0.42**	**1.49**	**[0.94;2.03]**				

*σ_v_*	1.8	1.7	0.11	[–0.17;0.4]				

\[ \sigma_{t_{0}} \]	0.12	0.12	0.12	[–0.18;0.42]				

*σ_w_*	0.1	0.13	0.27	[–0.1;0.65]				

	**INT CONGRUENT**				**INT INCONGRUENT**			

*a*	1.42	1.41	0.03	[–0.18;0.24]	1.39	1.46	0.13	[–0.03;0.28]

***v***	4.71	4.82	0.07	[–0.23;0.37]	**4.46**	**5.08**	**0.38**	**[0.13;0.63]**

*t_0_*	0.32	0.31	0.16	[–0.01;0.32]	0.32	0.32	0.07	[–0.06;0.2]

*w*	0.41	0.4	0.07	[–0.19;0.34]	0.37	0.35	0.14	[–0.09;0.37]

***σ_v_***	1.46	1.5	0.03	[–0.25;0.3]	**1.37**	**1.69**	**0.22**	**[0.01;0.44]**

\[ \sigma_{t_{0}} \]	0.12	0.11	0.09	[–0.16;0.35]	0.14	0.14	0.02	[–0.22;0.26]

***σ_w_***	0.12	0.12	0	[–0.37;0.38]	0.11	0.12	0.1	[–0.23;0.43]

	**TS COLOR**				**TS FORM**			

***a***	**1.63**	**1.86**	**0.38**	**[0.08;0.68]**	1.85	1.83	0.03	[–0.19;0.26]

***v***	**3.89**	**5.04**	**0.69**	**[0.32;1.05]**	**3.53**	**4.11**	**0.37**	**[0.07;0.66]**

***t_0_***	**0.34**	**0.3**	**0.69**	**[0.44;0.93]**	**0.34**	**0.3**	**0.56**	**[0.31;0.8]**

***w***	**0.38**	**0.44**	**0.48**	**[0.11;0.84]**	0.38	0.37	0.08	[–0.22;0.38]

***σ_v_***	1.61	1.71	0.08	[–0.25;0.42]	1.4	1.41	0	[–0.29;0.29]

\[ \sigma_{t_{0}} \]	0.14	0.14	0.04	[–0.31;0.38]	0.15	0.15	0.05	[–0.27;0.37]

***σ_w_***	**0.1**	**0.14**	**0.48**	**[0.12;0.83]**	0.11	0.11	0.08	[–0.29;0.44]


Note. Significant differences are highlighted using bold font. VS = visual search paradigm; 2CRT = 2CRT paradigm; Int = interference paradigm; TS = task-switching paradigm.

### Discussion of model-based distributional analyses

Like the results for the descriptive distributional analyses, the results from the model-based distributional analyses also revealed similarities and differences in the nature of sequential effects on RT distributions. For most paradigms, the drift rate *v* was sensitive to the sequential effects consistently across models, with a faster accumulation of evidence in repetition trials compared to alternation trials. Additionally, the time for stimulus encoding and response execution *t_0_* differed between repetition and alternation trials (i.e., larger *t_0_* for alternation trials) for the two tasks of the task-switching paradigm and the color targets in the visual search paradigm. In contrast, for the 2CRT paradigm the largest effects occurred on the relative starting point *w*, although the direction of the effect was not consistent across models.

Based on results from previous studies (Burnham, 2018; Schmitz & Voss, 2012) and the standard interpretation of the diffusion model parameters, it seems plausible that sequential effects map onto the drift rate *v* and the non-decision time *t_0_*. Effects on the drift rate *v* were also observed previously in the task-switching paradigm (Schmitz & Voss, 2012) and visual search paradigm (Burnham, 2018), and these would indicate that the sequential effect arises partly due to changes in the decision making process. A higher drift rate *v* in repetition trials compared to alternation trials, which is the pattern we mostly observed except for the 2CRT paradigm in the four-parameter and seven-parameter diffusion models, could be interpreted as a speeded uptake of information (Ratcliff, 2014) due to a pre-activation of, for example, the target dimension in the visual search paradigm, the target category in the 2CRT paradigm, the task-set in the task-switching paradigm, or event files for all paradigms.

An effect on the non-decision time *t_0_*, on the other hand, locates sequential effects outside the decision process and rather into processes such as stimulus encoding and response execution (Ulrich et al., 2015). A larger non-decision time for alternation trials compared to repetition trials could, for example, reflect attentional shifts on alternation trials, with participants having to shift attention to the relevant dimension or task before information accumulation could begin. This would suggest that sequential effects result from processing system adjustments made before the start of evidence accumulation in the current trial (e.g., Rogers & Monsell, 1995; Wolfe, 1994). On this account, the lack of sequential effect on *t_0_* in the VS form condition may have been a Type II error as this effect was also reported by Burnham (2018) for the visual search paradigm. Furthermore, the absence of this effect in the interference task suggests—like the small effect on mean RT—that there is no major reconfiguration of the processing system based on the change in congruency status, although the effect may simply have been too small to pick up.

Alternatively, changes in the non-decision time could also reflect deactivating the stimulus-response mapping from the previous trial before executing the current response as is suggested, for example, by the binding hypothesis for partial repetition costs (Hommel, 1998, 2004). This would suggest that sequential effects arise after the decision process. In our case, partial repetition costs—that is, RT costs that occur because of a repetition of the response whereas the identity of stimulus switches—could arise in alternation trials of the visual search and the task-switching paradigm. However, in the 2CRT paradigm in which the response always changes with a change in the stimulus type and in the interference paradigm for which we implemented a minimized confound design alternating in odd and even trials between different stimuli, partial repetition costs should not occur at all. This fits nicely with the observation that *t_0_* differs mainly for the visual search paradigm and the task-switching paradigm in the four-parameter and seven-parameter diffusion model.

For the 2CRT paradigm, effects on the relative starting point *w* would also be plausible as a repetition of the response category always goes along with a repetition of the response itself. Thus, an increased bias towards the correct response in repetition trials which we find in the four-parameter and seven-parameter diffusion model is equivalent to a bias towards the response of the previous trial which would reflect an automatic response activation as source of facilitation (Soetens et al., 1985). The same mechanism was introduced in the model by Goldfarb et al. (2012) and was demonstrated to be well able to explain even higher-order sequential effects. As briefly mentioned above, the direction of the effect should be consistent independent of the exact model version. However, for the EZ model we find the reversed effect.

Changes in *w* are not plausible for the paradigms other than 2CRT, however. In these other paradigms, the correct response either repeated in both repetition and alternation trials (e.g., in the visual search paradigm) or was orthogonal to the definition of repetition and alternation trials (as in the task-switching paradigm). Thus, there was no systematic difference between repetition and alternation trials with respect to the identity of the correct response, and hence no way for the system to be biased more toward the correct response for repetitions than for alternations, as would normally be the standard pre-activation-based interpretation of an effect on *w* in the diffusion model (e.g., Ratcliff & McKoon, 2008). Furthermore, for the task-switching paradigm the direction of the effect also depended on the exact diffusion model, with the EZ model again even showing an alternation advantage.

The effect on the boundary separation *a* also seems very implausible, because *a* is typically interpreted as the speed-accuracy trade-off set at the beginning of an experimental block or trial (Voss et al., 2004). However, whether a trial is a repetition or an alternation trial cannot be known before stimulus onset. In most paradigms, participants should be able to tell whether the current trial is a repetition or an alternation trial only during or even after the decision process. However, the boundary separation should already have been set before this point in time. Furthermore, the direction of the effect is also against typical expectations. In most cases, except for the four-parameter and seven-parameter diffusion model for the 2CRT paradigm, there seems to be a higher response caution in repetition trials, which is not predicted by most theoretical accounts of sequential effects (e.g., Botvinick et al., 2001; Wolfe, 1994). These observations clearly question whether the diffusion model is suitable for describing sequential effects.

Another remarkable feature of the results of all three diffusion models is that the different tasks and conditions with larger sequential effects on mean RT clearly showed significant effects on more model parameters compared to conditions with smaller sequential effects. Only the form task in the task-switching paradigm shows quite large task-switching costs (see Table 1) with few parameters sensitive to this effect. Additionally, for some conditions in which there was a significant effect on the mean RTs but no significant effect on accuracy (as indicated by the 95% confidence intervals of the delta accuracies; e.g., congruent stimuli of the interference paradigm), all three models failed to detect a difference in any parameter. In contrast, in conditions in which the accuracy differed significantly but there was no significant sequential effect on mean RTs (e.g., form targets of the visual search task), the models (except for the seven-parameter diffusion model) were well able to capture the sequential effects.

Taken together, these observations could mean that depending on task and stimulus characteristics, repetitions versus alternations differ in many different ways (i.e., corresponding to the different parameters that capture the sequential effects) or not at all. Alternatively, repetitions versus alternations could differ with respect to some process that is not really captured by the model (i.e., the model could be inappropriate for modelling sequential effects), and these many parameter changes as well as inconsistencies across and within paradigms could reflect the model’s best attempt to fit distributions that were actually generated in accordance with some other model. The examination of the sequential effects on specific parameters in specific conditions above seems to favor the latter possibility. Another test for the adequacy of a model is its actual fit to the data to which we will turn in the next section.

### Model fits across paradigms

**Figure A1:**
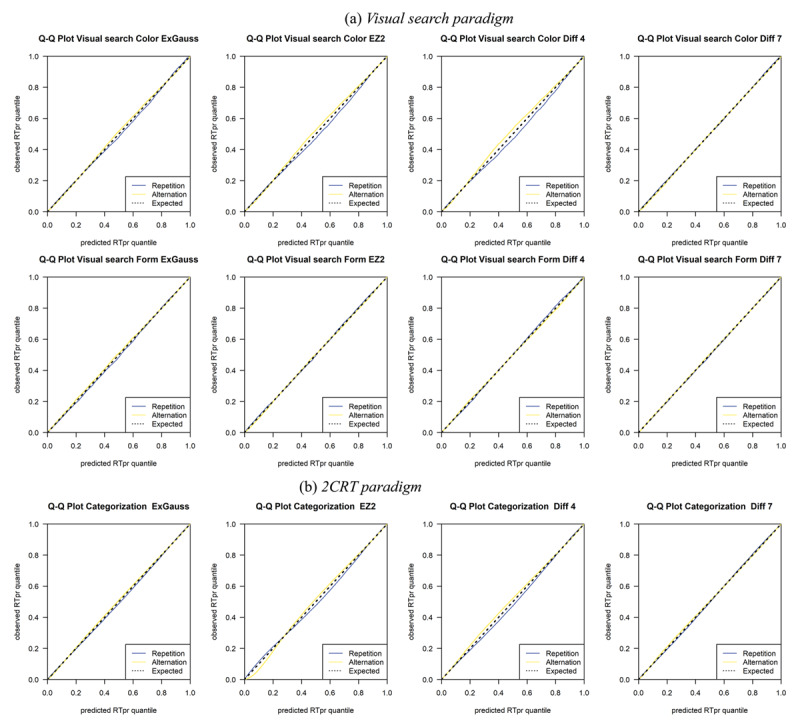
Quantile-quantile plots of the predicted and observed percentile ranks (\[
RT_{\rm pr}
\]) separately for each model – the ex-Gaussian (ExGauss), the EZ model (EZ), the four-parameter diffusion model (Diff 4), and the seven-parameter diffusion model (Diff 7) – for (a) the color (top) and form (bottom) targets of the visual search paradigm and (b) the 2CRT paradigm.

**Figure A2:**
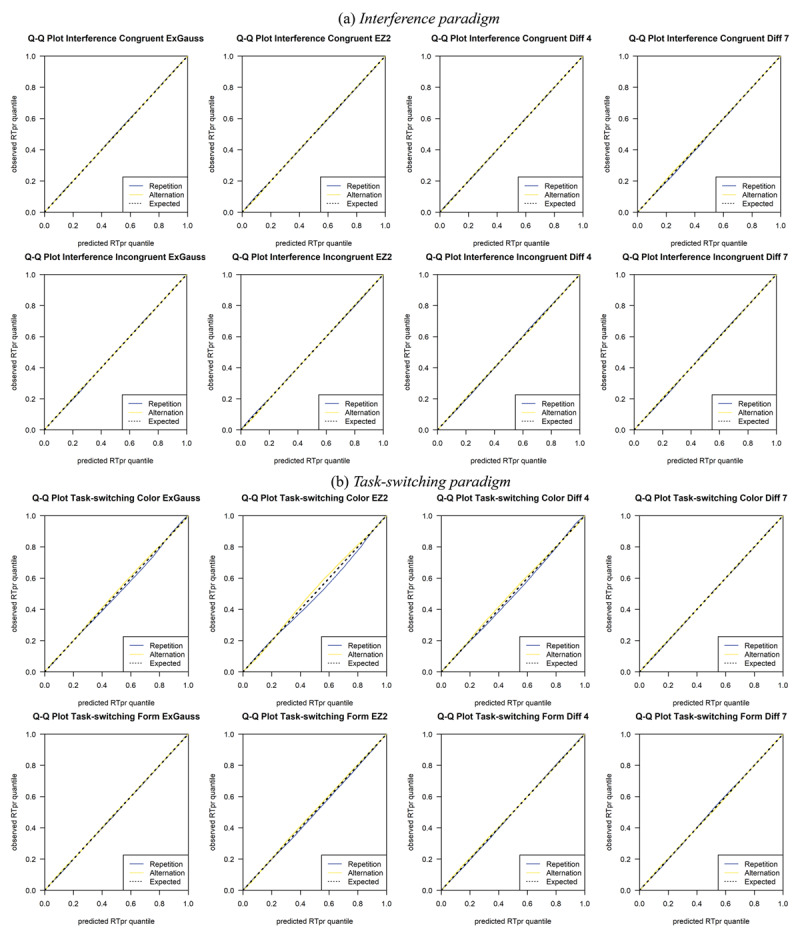
Quantile-Quantile plots of the predicted and observed percentile ranks (*RTpr*) separately for each model – the ex-Gaussian (ExGauss), the EZ model (EZ), the four-parameter diffusion model (Diff 4), and the seven-parameter diffusion model (Diff 7) – for (a) the congruent (top) and incongruent (bottom) stimuli of the interference paradigm and (b) the color (top) and form (bottom) task of the task-switching paradigm.

For the comparison of the model fits, we conducted a *χ*^2^-test that tested the deviation between predicted and observed number of trials per percentile for repetition and alternation trials in a combined test. Additionally, we computed a Kolmogorov-Smirnov test which considers the distance between the predicted and observed cumulative density distributions (CDF) and compares the maximum distance against a critical value, which must be done separately for the distributions obtained in repetition and alternation trials. We based both comparisons on pooled percentile ranks \[
RT_{\rm pr}
\] in order to get comparable distributions that could be combined across participants to maximize the sample size of each comparison. Therefore, we generated simulated RTs for each participant and for each model using the parameter estimates from the previous analyses. In a second step, we calculated the percentile ranks separately for each paradigm and each stimulus type to compare these predicted data with the originally observed data, and these comparisons are visualized in the Appendix in Figures A1 and A2. Please note that both tests only consider the goodness of the fit but do not include a penalty for model complexity in terms of numbers of parameters or its functional complexity.

#### Visual search paradigm

For the color targets in the visual search paradigm, the seven-parameter diffusion model captured the results best (see Table 6) in that both the *χ*^2^-test as well as the Kolmogorov-Smirnov test resulted in the smallest *χ*^2^ value and the smallest maximum CDF separation, with no significant differences between the observed and predicted percentile ranks. However, for the form targets within this paradigm the results are unclear. When evaluating the *χ*^2^-test, the ex-Gaussian distribution seems to capture the data best. In contrast, the seven-parameter diffusion model is favored when evaluating the results from the Kolmogorov-Smirnov test. Nevertheless, when considering only the results of the Kolmogorov-Smirnov test for the form targets, all models seem to describe the data quite well as there are no significant deviations between the observed and predicted percentile ranks. This is also depicted in the Appendix in Figure A1. And, as can be seen in Figure A1 and in Table 6, the models have more difficulties reproducing the data from the color targets than from the form targets.

**Table 6:** Results of the *χ*^2^-test computed jointly for alternation and repetition trials as well as from the Kolmogorov-Smirnov test computed separately for alternation and repetition trials for each paradigm, stimulus type, and model.


	*χ*^2^(99)(*P*)	*D_A_*(*P*)	*D_R_*(*P*)	*χ^2^*(99)(*P*)	*D_A_*(*P*)	*D_R_*(*P*)

	VS COLOR			VS FORM		

**ExGauss**	136(.008)	0.0179(.056)	0.0177(.056)	**108** **(**.246**)**	0.0072(.919)	0.0077(.912)

EZ	185(< .001)	0.0341(< .001)	0.034(< .001)	127(.029)	0.0113(.444)	0.0121(.412)

Diff4	175(< .001)	0.0274(< .001)	0.0269(.001)	114(.143)	0.0085(.789)	0.0091(.768)

**Diff7**	**100** **(**.467**)**	**0.0092** **(**.729**)**	**0.009** **(**.744**)**	109(.232)	**0.0062** **(**.979**)**	**0.0069** **(**.958**)**

	**2CRT**					

**ExGauss**	**184** **(<.001)**	**0.011** **(.001)**	**0.0111** **(.001)**			

EZ	**1494**(< .001)	0.0252(< .001)	0.0256(< .001)			

Diff4	299(< .001)	0.0228(< .001)	0.0232(< .001)			

Diff7	244(< .001)	0.0151(< .001)	0.0153(< .001)			

	**INT CONGRUENT**			**INT INCONGRUENT**		

**ExGauss**	**103** **(.371)**	0.0038**(.973)**	0.0038**(.974)**	**123** **(.051)**	0.004**(.959)**	0.0039**(.967)**

EZ	139(.005)	0.0081(.251)	0.0079(.266)	155(< .001)	0.0072(.378)	0.0073(.362)

Diff4	117(.101)	0.0085(.204)	0.0083(.211)	138(.006)	0.0086(.187)	0.0086(.186)

Diff7	112(.170)	0.0075(.331)	0.0074(.338)	131(.018)	0.0069(.425)	0.0069(.436)

	**TS COLOR**			**TS FORM**		

**ExGauss**	196(< .001)	0.0124(.015)	0.0124(.015)	**110** **(.219)**	**0.0028** **(>.999)**	**0.003** **(.999)**

EZ	344(< .001)	0.0268(< .001)	0.0267(< .001)	128(.027)	0.0083(.227)	0.0083(.231)

Diff4	250(< .001)	0.0175(< .001)	0.0175(< .001)	142(.003)	0.0119(.023)	0.0123(.018)

**Diff7**	**127** **(**.032**)**	**0.0039** **(.972)**	**0.0039** **(.969)**	**120** **(.072)**	**0.0075** **(.332)**	**0.0076** **(.325)**


Note. VS = visual search paradigm; 2CRT = 2CRT paradigm; Int = interference paradigm; TS = task-switching paradigm; ExGauss = ex-Gaussian distribution; EZ = EZ-diffusion model; Diff4 = four-parameter diffusion model, Diff7 = seven-parameter diffusion model.

#### 2CRT paradigm

Although for the 2CRT paradigm all models showed a significant deviation between observed and predicted percentile ranks, a visual inspection (see Appendix) shows that the fits were actually reasonably good for each of the models. Comparing the test values in Table 6, the ex-Gaussian distribution seems to describe the data best while the EZ model showed the greatest deviation between the predicted and observed percentile ranks.

#### Interference paradigm

As for the 2CRT paradigm, the ex-Gaussian distribution seems to describe the data from the interference task best (see Table 6). But again, the different Kolmogorov-Smirnov tests detected no significant differences between the observed and predicted percentile ranks of any model. The *χ*^2^ test, in contrast, revealed some significant deviations between predicted and observed percentile ranks. Nevertheless, having a look at Figure A2 in the Appendix, all models seem to describe the data quite well.

#### Task-switching paradigm

Finally, for the task-switching paradigm, the ex-Gaussian distribution described the data from the form task best whereas the seven-parameter diffusion model described the data from the color task best (see Table 6). Overall, visual model fits (see Appendix) reveal that all models seem to fit the data quite well, except for small deviations in the predictions for the EZ model in the color task.

## General Discussion

The present study aimed to compare changes in RT distributions for repetition and alternation trials in different paradigms to see whether sequential effects show similarities beyond the level of mean RTs and accuracy. Furthermore, we were interested in whether stochastic RT models would be able to account for the sequential effects in different paradigms with similar mechanisms. Therefore, we investigated sequential effects on RT distributions in the visual search paradigm, the 2CRT paradigm, the interference paradigm, and the task-switching paradigm, using both descriptive and model-based analyses of RT distributions. The results of both approaches revealed that there are similarities between paradigms in distribution-level sequential effects, with some paradigms sharing more commonalities than others. The analyses additionally revealed, however, that there are also differences in these distributional effects not only between paradigms but even between stimulus types within the same paradigm.

One striking finding was that these clear differences between—and even within—paradigms in sequential effects already occurred at the level of mean RT and accuracy, as can be seen in Table 1. Sequential effects were on the order of 100 ms in the task-switching paradigm, 35 ms in the 2CRT paradigm, and only 10 ms in the interference paradigm. Perhaps most surprisingly, mean sequential effects in the visual search paradigm depended strongly on the target dimension, with color targets benefiting from a dimension repetition by about 45 ms, whereas form targets showed virtually no repetition benefit at all. Furthermore, where sequential effects on mean RT were small or absent (i.e., visual search for form and interference), examination of the Vincentized distributions and \[
RT_{\rm pr}
\] suggested that there were virtually no sequential effects anywhere in the distributions—not just on the means. Clearly, examination of the similarities and differences of sequential effects on RT distributions can be most revealing for those conditions where strong effects were found in the first place—that is, the visual search for color, 2CRT, and task-switching paradigms.

Similarities between paradigms were present, for example, in the effects of repetition versus alternation trials on the parameter estimates with which the ex-Gaussian distribution as well as the diffusion model described the RT distributions. The *μ* parameter of the ex-Gaussian distribution differed between repetition and alternation trials for all paradigms, reflecting sequential effects that shifted the RT distribution. Furthermore, for most paradigms the diffusion model was sensitive to sequential effects in the drift rate parameter *v* which is typically interpreted as a difference in the speed of information uptake (Voss et al., 2004), or *t_0_* the time for stimulus encoding and response execution. These results replicate nicely findings from previous applications of the diffusion model to analyze sequential effects in the task-switching paradigm (Schmitz & Voss, 2012) and the visual search paradigm (Burnham, 2018).

Differences between paradigms and stimulus types were already evident in the shapes of the RT distributions of repetition and alternation trials assessed via Vincentized RTs and \[
RT_{\rm pr}
\]. Different patterns occurred for different groups of paradigms. For color targets in the visual search paradigm and for both stimulus dimensions in the task-switching paradigm, sequential effects tended to increase in the higher quantiles of the RT distribution. This is depicted by rather monotonic decreases and increases in frequency across \[
RT_{\rm pr}
\] values for repetition and alternation trials, respectively. For the 2CRT paradigm which also showed clear sequential effects, however, sequential effects appeared to be relatively constant across quantiles, if anything decreasing at the highest quantiles. The latter is represented in the \[
RT_{\rm pr}
\] by the response frequencies for repetition and alternation trials that nearly converge in the higher percentile ranks. And finally, in the case of visual search form targets and the interference paradigm where the sequential effect on mean RT was smallest, there was also at most a small repetition advantage at the majority of quantiles paired with a small repetition disadvantage at the highest quantile.

The models we examined were also sensitive to these differences in the RT distributions of repetition and alternation trials. For example, as well as the *μ* that reflected changes in sequential effects in all paradigms, different additional parameters of the ex-Gaussian distribution differed between repetition and alternation trials depending on the paradigm and stimulus type. Different diffusion model parameters also varied significantly between repetition and alternation trials for different paradigms and stimulus types, with the latter effects being even more pronounced between stimulus types than between paradigms. However, the exact conclusions about the parameters of the diffusion model that were affected by sequential effects depended on the version of that model under consideration.

### Diffusion model as a tool to explain sequential effects

In our model-based distributional analysis, we used different variants of the diffusion model because the diffusion model previously successfully passed multiple validation checks (e.g., Arnold et al., 2015; Ratcliff & McKoon, 2008; Voss et al., 2004), and thus the parameters of this model are widely interpreted in terms of cognitive processes. However, in our investigations we observed some irregularities that are difficult to reconcile with standard interpretations of its parameters in terms of processes.

First, the observations that differences between the RT distributions of repetition and alternation trials differ more strongly between stimulus types than between paradigms and even seem to depend on the size of the sequential effects in mean RTs is rather surprising. Both observations might have the same source, because in most paradigms one stimulus type showed a larger sequential effect than the other. Initial exploratory analyses revealed that these observations might be grounded in the specification of the model structure. Analyzing the variance-covariance matrix between the four main parameters of the seven-parameter diffusion model using the Fisher-information matrix revealed medium to high correlations between parameters. This suggests that the different parameter estimates considered as criteria in the *t*-tests are not independent, so large differences in one parameter might be associated with large differences in another parameter. Because of the high correlations among parameters, it is difficult to say which parameters are causally involved in the sequential effect and which are only incidentally involved in the effect. And, if the fit of the diffusion model does not really reveal which parameters are affected, then the model does not help to identify which cognitive processes are affected, even given the usual assumptions about which parameters are associated with which cognitive processes.

Furthermore, we observed a second irregularity—namely, that for some paradigms, parameters that most certainly should not be affected by the present sequential manipulation were sensitive to sequential effects. Two examples are the boundary separation *a* and the relative starting point *w*, which could not logically differ for repetition versus alternation trials for any paradigm except the 2CRT task. These effects are clearly inconsistent with the usual interpretations of these parameters within the diffusion model, and they thereby weaken the interpretations of effects on its parameters.

It is also noteworthy that the conclusions drawn for the present research question depended on the exact version of the diffusion model. Technically, the seven-parameter and four-parameter diffusion models are different models, so differences in the parameters that were sensitive to sequential effects might be simply explained by these different model structures. These models do share many assumptions, however, and their common parameters are generally interpreted in the same ways, making the sequential effects on different parameters somewhat puzzling. Moreover, such differences were also present for the EZ model versus the four-parameter diffusion model, which should be essentially the same model with only differences in the procedures for parameter estimation. Whereas the parameters of the EZ model are estimated via equations for which parameter search ends once a solution is found with a mean squared error being equal to 0, for the four-parameter diffusion model we used maximum-likelihood estimation. And, as had already been found in previous studies (Wagenmakers et al., 2007), search routines for maximum-likelihood estimation again had difficulties converging on realistic parameter estimates for participants with low error rates, for both the seven-parameter as well as the four-parameter diffusion model. Thus, the differences in the conclusions might result from diverging estimation routines.

However, for the paradigms under consideration low error rates are a very common observation. And still the interpretability of the parameters should be ensured, especially because we observed those violations also in the 2CRT task, a paradigm frequently used to validate the diffusion model (see, e.g., Ratcliff & McKoon, 2008). Therefore, the diffusion model accounts of sequential effects look less promising when comparing multiple paradigms than when only a single paradigm was considered (e.g., Burnham, 2018; Schmitz & Voss, 2012).

There do exist other stochastic models for RT performance such as, for example, the linear ballistic accumulator model (Brown & Heathcote, 2008), the parallel grains model (Miller & Ulrich, 2003), and the cascade model (McClelland, 1979). But just like the diffusion model, most of these models assume for simplicity that the decision maker is in the same state at the beginning of each trial, a condition that is clearly violated due to the emergence of sequential effects. Nevertheless, as the diffusion model seems to have difficulties describing sequential effects, it might be worthwhile to evaluate in future research which other stochastic models provide more plausible descriptions of sequential effects. This might help to identify processes that are sensitive to sequential effects and to include these into stochastic RT models elaborated to describe trial-to-trial variations in performance.

Nevertheless, the visual inspection of the model fits (see Appendix) reveals that the diffusion model is still able to mimic the observed changes in the RT distributions for the present paradigms. Thus, although the results of the present study reveal that the parameters of the diffusion model are hard to interpret in terms of processes influenced by sequential effects, they still might provide some descriptive information for our present research question, namely whether sequential effects also share commonalities when considering the changes in their RT distributions.

### Implications concerning sequential effects

The analogous changes in the RT distributions of repetition versus alternation trials across paradigms observed in the present study strengthen the idea of sequential effects operating on the same cognitive mechanisms across paradigms. Assuming common cognitive mechanisms as is, for example, considered in the framework of binding and retrieval in action control (Frings et al., 2020), one would expect that responses in repetition and alternation trials are affected similarly irrespective of the exact paradigm. In our study these similarities are reflected in similar changes in parameter values in models such as the ex-Gaussian distribution. These analogous changes are also in line with our theoretical review in the introduction. There, we observed that theoretical accounts across paradigms do share some assumptions. These include the role of strategic expectations, the contribution of (implicit) memory, and the role of cognitive conflict. Thus, the present results provide encouragement that it would be informative to explore the commonalities across paradigms of sequential effects that could be expected based on these accounts.

There were also fairly clear differences in the distributional changes between repetition and alternation trials across paradigms. These were reflected in, for example, differences between paradigms in sequential effects on the *σ* and *τ* parameters of the ex-Gaussian distribution, as well as in the different sequential effect on the shapes of the \[
RT_{\rm pr}
\] and the Vincentized distributions. That repetitions and alternations affect RT distributions differently across paradigms suggests that there are task-specific sequence-sensitive processes in addition to common cognitive mechanisms. These task-specific mechanisms in sequential effects highlight that caution is required when generalizing results from sequential effects of one paradigm to another paradigm, as our results reveal that not all conclusions can be generalized across paradigms. Rather, our observations of both shared distributional changes as well as paradigm-specific distributional changes demonstrate the importance of investigating mechanisms in several paradigms, especially when testing comprehensive theoretical accounts that should be applicable across multiple paradigms.

Including several paradigms in single studies would also help to distinguish which exact mechanisms are shared between paradigms and in which mechanisms paradigms might differ. Our results suggest that sequential effects of some paradigms have more in common than other paradigms. For example, the distributional changes of the visual search paradigm for color targets as well as the task-switching paradigm seemed to show more similarities than, for example, the distributional changes within the 2CRT paradigm. It is currently unclear which processing mechanisms these paradigms share, because the similarities and differences between paradigms are seldom investigated, although this could greatly increase our understanding of fundamental cognitive processes.

Finally, our results mainly leave open the questions regarding the differing nature of sequential effects across stimulus types within each paradigm. For example, RT distributions of repetition versus alternation trials seem to behave differently for color targets and form targets of the visual search paradigm. While it is common to assume paradigm-specific cognitive mechanisms for sequential effects, stimulus-specific effects have rarely been considered. Therefore, it would be interesting for future research to investigate differences in stimulus materials as well as differences in paradigms. Ideally, this could be done using within-participant designs in which both paradigm-specific and stimulus-specific effects could be quantified.

### Conclusion

The picture of sequential effects emerging from the present study is quite complex. Looking at these effects at a detailed distributional level and comparing them across multiple experimental paradigms provides a much richer picture of the effects than can be obtained by looking at mean RTs within a single paradigm. This richer picture correspondingly offers a much broader data base—and thus much greater challenges—to theoretical models designed to account for the effects. The results thus provide a further demonstration that distinctive features of RT effects are lost when only mean RTs are analyzed, and they further suggest that studying the commonalities and differences of these distinctive features across multiple paradigms can provide further insight and modelling challenges not only for the sequential effects studied here but for almost all effects that are present across multiple paradigms.

## Data Availability

All data, analysis scripts, and scripts to run the experiment are publicly available on the open science framework (https://osf.io/p6v9z/).

## Footnotes

In most cases sequential effects in interference tasks are referred to as “congruency sequence effects” (Botvinick et al., 2001; Gratton et al., 1992; Hommel, 1998). Those describe the observation of a larger congruency effect after a previously congruent trial compared to a previously incongruent trial. This is just a different way of framing a sequential effect analogous to the ones already described, however, because it again refers to an interaction between congruency in trial *n* – 1 and congruency in trial *n*.

We used the standard model specifications described in the introduction with two exceptions: In contrast to the most common procedure, we included error trials for fitting the ex-Gaussian distribution to consider the same subset of trials in all models. Also, for the EZ model, we considered the model from Grasman et al. (2009) including the estimation of a biased starting point. To make the investigation of the starting point easier across the different diffusion models and the different paradigms, we always considered the starting point relative to the criterion *a* instead of an absolute starting point.

For the ex-Gaussian and the EZ model we programmed the likelihood/error function ourselves. However, for the computation of the likelihoods for the diffusion model we used the WienR package (Hartmann & Klauer, 2021).

The number of replications differed between the models because of the time needed for fitting the models. For the ex-Gaussian distribution and the EZ model, the time to fit the model is negligible. However, fitting the four- and seven-parameter diffusion models is much more time consuming. Therefore, we evaluated after each ten replications whether the conclusions drawn would differ in two consecutive fits to decide whether more replications were necessary. Because they already seemed to be stable after ten replications, we stopped at this point for these two models. Furthermore, for the EZ model the error term per participant and condition needs to be equal to 0 to ensure a valid estimation of parameters. Except for two observations for the color targets in the visual search paradigm, error terms were very close to 0 (being smaller than 3.8 × 10^–9^). We conducted the analysis also without these observations, but as the exclusion did not change something in the conclusions, we decided to report the complete results.

As the first bin for the interference paradigm with above chance accuracy still had a cumulative error rate of 49%, we decided to be more conservative and chose the lower boundary of the second bin as criterion for trial exclusion.

## Appendix A

Below, we included a visual representation of the excellent fit of the ex-Gaussian, the EZ model, the four-parameter diffusion model, and the seven-parameter diffusion model the observed RTs of each paradigm and stimulus type.
